# Characterizing conflict and congruence of molecular evolution across organellar genome sequences for phylogenetics in land plants

**DOI:** 10.3389/fpls.2023.1125107

**Published:** 2023-03-30

**Authors:** Alexa S. Tyszka, Eric C. Bretz, Holly M. Robertson, Miles D. Woodcock-Girard, Karolis Ramanauskas, Drew A. Larson, Gregory W. Stull, Joseph F. Walker

**Affiliations:** ^1^Department of Biological Sciences, University of Illinois at Chicago, Chicago, IL, United States; ^2^Sainsbury Laboratory, School of Biological Sciences, University of Cambridge, Cambridge, England, United Kingdom; ^3^Department of Biology, Indiana University, Bloomington, IN, United States; ^4^Germplasm Bank of Wild Species in Southwest China, Kunming Institute of Botany, Chinese Academy of Sciences, Kunming, Yunnan, China; ^5^Department of Botany, National Museum of Natural History, Smithsonian Institution, Washington, DC, United States

**Keywords:** phylogenetics, plastome, mitochondrial genome, chloroplast genome, phylogenomics, combinability, phylogenetic conflict

## Abstract

Chloroplasts and mitochondria each contain their own genomes, which have historically been and continue to be important sources of information for inferring the phylogenetic relationships among land plants. The organelles are predominantly inherited from the same parent, and therefore should exhibit phylogenetic concordance. In this study, we examine the mitochondrion and chloroplast genomes of 226 land plants to infer the degree of similarity between the organelles’ evolutionary histories. Our results show largely concordant topologies are inferred between the organelles, aside from four well-supported conflicting relationships that warrant further investigation. Despite broad patterns of topological concordance, our findings suggest that the chloroplast and mitochondrial genomes evolved with significant differences in molecular evolution. The differences result in the genes from the chloroplast and the mitochondrion preferentially clustering with other genes from their respective organelles by a program that automates selection of evolutionary model partitions for sequence alignments. Further investigation showed that changes in compositional heterogeneity are not always uniform across divergences in the land plant tree of life. These results indicate that although the chloroplast and mitochondrial genomes have coexisted for over 1 billion years, phylogenetically, they are still evolving sufficiently independently to warrant separate models of evolution. As genome sequencing becomes more accessible, research into these organelles’ evolution will continue revealing insight into the ancient cellular events that shaped not only their history, but the history of plants as a whole.

## Introduction

Plant cells harbor two organelle types that each contain their own genetic material: plastids (typically chloroplasts) and mitochondria. While the mitochondrial genome has been used widely in animal phylogenetics (e.g., Lavrov, 2007), in plants, the plastome has been the primary genomic resource for phylogenetic studies over the past 35 years (Palmer et al., 1988; Moore et al., 2007; Moore et al., 2010; Soltis et al., 2011; Smith and Brown, 2018). The widespread use of plastid genes and genomes, however, has largely been motivated by practical considerations (e.g., the absence of paralogy, ease of PCR amplification, rates of evolution useful for reconstructing deep relationships; Clegg, 1993). Over the past decade, new sequencing technologies and protocols have facilitated the increased use of nuclear data for phylogenomic investigations of plants and other major branches on the tree of life (e.g., Dunn et al., 2008; McKain et al., 2012; Weitemier et al., 2014; *One Thousand Plant Transcriptomes Initiative, 2019*). Nevertheless, the plastome will likely remain a critical source of phylogenetic information, given that its typically uniparental mode of inheritance results in a unique evolutionary history that, in combination with nuclear phylogenies, is valuable for detecting both recent and ancient hybridization (Rieseberg and Soltis, 1991). Additionally, in species with maternal inheritance, it can be used to investigate the contributions of seed dispersal to phylogeographic patterns (Asmussen and Schnabel, 1991; McCauley, 1994). Because of the central importance of chloroplasts in photosynthesis, studying the plastome can also provide insights into this important cellular process. In plants that have lost the ability to photosynthesize, including parasitic species, the plastome often shows major structural changes, gene loss, and high rates of pseudogenization, reflecting greatly reduced evolutionary constraint on the plastome in these species (e.g., Petersen et al., 2015; Schneider et al., 2018; Qu et al., 2019).

Mitochondrial genomes (like plastomes) tend to be uniparentally inherited in most plants and, therefore, it is generally expected that mitochondrial phylogenies should show concordance with those of the plastome. This assumption arises based on the expectation that the organelles are inherited from the same parent, which may not always be the case. Indeed, there are many plant lineages in which biparental inheritance of at least one organelle is common and also those in which mitochondrial genomes and plastomes are usually inherited from different parents (Mogensen, 1996; Camus et al., 2022). There has also been recent evidence that patterns of biparental inheritance may vary with environmental conditions in some taxa (Chung et al., 2023).

The molecular evolution of mitochondrial genomes in seed plants shows remarkable differences from that of plastomes, with the former exhibiting slower substitution rates, more structural evolution, a greater tendency to uptake foreign DNA, and significantly greater variation in size (Wolfe et al., 1987; Alverson et al., 2010). Although multiple studies have compared phylogenetic signal from subsets of plastid and mitochondrial genes (e.g., Qiu et al., 1999; Barkman et al., 2000; Bowe et al., 2000; Chaw et al., 2000; Qiu et al., 2010), a detailed comparison of plastid and mitochondrial phylogenies across seed plants has not been undertaken at a genome scale. Plant mitochondrial genome evolution has been studied by numerous researchers (e.g., Palmer et al., 2000; Alverson et al., 2010; Knoop et al., 2011; Mower et al., 2012; Zervas et al., 2019), but the challenges associated with mitochondrial genome assembly, as well as the limited historical use of mitochondrial sequences in plant phylogenetics, have resulted in a relative dearth of complete mitochondrial genomes (ca. ~400) compared to the abundance of complete plastomes (~9000) publicly available on GenBank (accessed July 19, 2022).

Given that high-throughput sequencing technologies now make sequencing of both organellar genomes a relatively easy task, including from “off-target” reads and low coverage genome skimming, it is worth considering whether mitochondrial genomes should be more broadly integrated into plant evolutionary and phylogenomic studies (Weitemier et al., 2014; Cai et al., 2022). More fundamentally, the extent of evolutionary concordance between the two organelles remains unknown, not only in terms of their supported phylogenetic topologies, but also in their rates of molecular evolution and sequence composition among genes and across the land plant phylogeny. Do plastid and mitochondrial genomes show strongly supported differences in regions of topological conflict across land plants, or are the differences largely confined to areas of poor support/resolution? Do genes in plastid and mitochondrial genomes tend to evolve similarly, such that they can be considered to share an evolutionary model, or should their evolution be modeled separately? If the latter is the case, what molecular evolutionary properties (e.g., rate or rate heterogeneity) tend to vary between these genomes? Do shifts in compositional bias tend to occur at the same branches in the land plant phylogeny, suggesting a shared evolutionary response to selective pressures, gene conversions, and mutation biases (Eyre-Walker and Hurst, 2001; Lynch and Walsh, 2007) and potentially explaining conflict across the genomes (Smith et al., 2022)? Recent studies (e.g., Gonçalves et al., 2019; Walker et al., 2019; Zhang et al., 2020) have provided valuable insight into the sources of conflict and concordance in phylogenetic signals among genes within the plastome. To address the questions outlined above, we build upon this work to investigate major patterns of plastome and mitochondrial genome evolution within the context of land plant phylogeny, leveraging a newly compiled dataset that includes all land plant (Embryophyta) species with available complete genomes from both organelles.

## Materials and methods

### Dataset acquisition and curation

Plastid and mitochondrial genomes were downloaded from the National Center for Biotechnology Information (NCBI) GenBank database, using search terms “plants”, “biomol_genomic”, “refseq”, “is_nuccore” and either “plastid”, “chloroplast”, or “mitochondrion” depending upon the organelle. Associated biological information, such as organism name, accession number, organelle source, TaxID, and sequence length were retrieved using novel scripts (https://github.com/ericbretz/chloro-mito-phylo), which leveraged functions from the Python 3 library Biopython v1.79 (Cock et al., 2009). To create a unique set of mitochondrial genomes, only the longest sequence was retained for cases of duplicate TaxIDs. To construct the plastome set, sequence records annotated as either “chloroplast” or “plastid” were treated as the same organelle. Similar to the mitochondrial set, duplicates were handled by retaining only the longest sequence. Algal sequences were removed from the set due to the difficulty of verifying homology by sequence similarity, leaving only land plant sequences. Finally, only sequences from the taxa present in both the mitochondrial and the plastid datasets were retained. Land plant species with available sequences for both genomes can be found in **Supplementary Table 1**.

Next, annotated open reading frames (ORFs) were extracted for each dataset. Any ORFs with no gene name (labeled as “hypothetical protein” or “orf”) were discarded. The sequences for each dataset were then clustered using VSEARCH v2.14.1 (Rognes et al., 2016) with options “–iddef 1 –id 0.5” to address potential annotation issues, such as differing naming schemes. Clusters were named after the most frequent sequences they contained. Any taxa that exhibited potential problems with the gene annotations were removed, resulting in a final taxon list for downstream analysis (**Supplementary Table 2**). Each cluster of nucleotide ORFs was treated as a set of orthologs and codon-aligned by first aligning amino acids with MAFFT v7.490 (Katoh and Standley, 2013), using options “–maxiterate 100000 –localpair –op 1.53 –ep 0 –bl 62”, then converting the alignment back to nucleotides using the translation align feature in Geneious v2022.2.2 (Kearse et al., 2012). Any sequences exhibiting significant differences from others in the alignment were removed. In a few cases, annotated ORFs included adjacent loci; the adjacent regions were removed with Geneious. Alignments from which any sequence was removed were re-aligned as described above. The alignments before and after the removal procedure as well as a novel script used to determine occupancy of the final dataset are available from GitHub (https://github.com/alexatyszka/phylorganelles). Final sampling consisted of 226 taxa from across land plants. The sampling contained 35 bryophytes: 11 liverworts, four hornworts, and 20 mosses; two species of ferns: *Psilotum nudum* and *Ophioglossum californicum*; four species of gymnosperms: *Cycas taitungensis*, *Ginkgo biloba*, *Pinus taeda*, and *Welwitschia mirabilis*; and 185 species of angiosperms, including two members of Nymphaeales and one member of Austrobaileyales. We did not include Amborellales in our sampling because the mitochondrial genome of Amborella has a known history of horizontal gene transfer; therefore, the ANA grade was represented by Nymphaeales and Austrobaileyales.

### Organelle tree inference and partition model testing

Three concatenated supermatrices containing either the plastome alignments (PLAST), the mitochondrial genome alignments (MITO), or both (COMB) were generated using *pxcat* from the package phyx v1.2 (Brown et al., 2017). For each of the three datasets, four maximum likelihood phylogenetic trees were inferred with different partitioning approaches using the GTR+I+G model of evolution, as implemented in IQ-TREE v1.6.12 (Nguyen et al., 2015; Chernomor et al., 2016; Nguyen et al., 2018). The first approach was an unpartitioned model, which resulted in the PLAST-Unpartitioned, MITO-Unpartitioned, and COMB-Unpartitioned phylogenetic trees. For the other three approaches, each gene was assigned its own model partition, with either the edge-equal (PLAST-Equal, MITO-Equal, and COMB-Equal trees), edge-proportional (PLAST-Proportional, MITO-Proportional, and COMB-Proportional trees) or edge-unlinked (PLAST-Unlinked, MITO-Unlinked, and COMB-Unlinked trees) partition model specified with the “-q”, “-spp”, and “-sp”, options, respectively. Under the edge-equal partition model, base transition frequencies for each partition are estimated separately, and all partitions share the same tree, including the same branch lengths. The edge-proportional model instead accommodates shifts in evolutionary rate among partitions by allowing each to have a tree with different branch lengths but requires that branch lengths are proportional across partitions (Chernomor et al., 2016). The edge-unlinked model has the most model parameters and allows evolutionary rates to vary freely among partitions, allowing the inferred tree of each partition to have completely different branch lengths, while only requiring that the trees share the same topology (Lopez et al., 2002). Tree inference for each combination of model and dataset was conducted with 1,000 ultrafast bootstrap2 (UFBoot) replicates to estimate support (Hoang et al., 2018). We measured each gene’s log-likelihood contribution to the total log-likelihood of the COMB-Proportional tree by constraining the topology to that of the COMB-Proportional tree and inferring the likelihood for each partition using the GTR model of evolution with the “-*wpl*” option implemented in IQ-TREE v.1.6.12.

### Test for combinability

In maximum likelihood tree estimation, a phylogenetic tree results from a combination of model parameter estimates, guided by a topology. The topology itself is not a model parameter, but does influence the values that some model parameters can take during tree optimization. We therefore refer to *trees* as having model parameters with estimated values (e.g., branch lengths, transition rates), whereas the *topology* refers only to the structure under which the parameters were estimated (i.e., the order of branching). We use the term *combinability* to mean that the two organellar genomes support a single tree rather than multiple trees based on information criteria scores (Neupane et al., 2019; Smith et al., 2020). We further extend this definition to describe the degree to which the data are consistent in terms of their molecular evolution as inferred by the optimal partitioning model.

The Bayesian Information Criterion (BIC) and corrected Akaike Information Criterion (AICc) scores for the twelve inferred PLAST, MITO, and COMB trees were obtained from IQ-TREE output files. To calculate BIC and AICc scores for models in which plastid and mitochondria sequences were analyzed under separate topologies, the log-likelihood values, the total number of aligned sites (*n*), and the number of parameters (*k*) relevant to the PLAST-Proportional (“-spp”) and the MITO-Proportional (“-spp”) or MITO-Unlinked (“sp”) trees were obtained from their respective IQ-TREE output files and were each summed together. This resulted in a log-likelihood value of −3034134.6942, an *n* of 162,101, and a *k* of 2,194, which were used to calculate the BIC and AICc scores.

A partitioning scheme was selected for the COMB dataset using PartitionFinder (Lanfear et al., 2012) as implemented in IQ-TREE with the option “-m MFP+MERGE”. A maximum likelihood tree was inferred using the best partitioning scheme and 1,000 UFBoot replicates (Hoang et al., 2018). The BIC and AICc scores for this tree (i.e., the COMB-Merged tree) were obtained from the IQ-TREE output file.

### Calculating Robinson-Foulds distance

All-by-all unweighted Robinson-Foulds (RF) distances (Robinson and Foulds, 1981) were calculated in a pairwise manner between all inferred plastome, mitochondrial genome, and combined phylogenies using the gophy program *bp* (https://github.com/FePhyFoFum/gophy). This was done with and without a support threshold (≥ 95% UFBoot). The R package igraph (Csardi and Nepusz, 2006) was used to infer a network of trees, in which each topology was a node and the inverse RF distances were the edge weights. Finally, the Fruchterman-Reingold algorithm (Fruchterman and Reingold, 1991) was used to construct the graph.

### Gene tree model testing for shifts in compositional heterogeneity

The rooted MITO-Proportional and PLAST-Proportional trees were used for testing whether individual genes evolved under mixed models of nucleotide composition. The respective input organelle tree was trimmed to match the taxon sampling of the gene in question using the program *pxtrt* from the phyx package (Brown et al., 2017). Testing for multiple models across a topology was performed using the program Janus (Smith et al., 2022) with the parameters “-rm -g -ue -ul -min 4”. Due to an as yet undiagnosed memory issue, model testing predictions could not be completed on the plastid gene *petG*. The pipeline for the procedure is available at https://github.com/gladshire/janus-model-shift.

### Dating, estimation of root-to-tip variance, and gene tree conflict analysis

The COMB-Merged tree rooted on the Bryophyte edge was converted to a chronogram using penalized likelihood (Sanderson, 2002) as implemented in treePL v1.0 (Smith and O’Meara, 2012). To estimate the optimal settings, we used the “prime” option in treePL and ran the full analysis with the settings set to “thorough”, “opt = 2”, “optad = 2”, “moredetailed”, and “optcvad = 2”. The minimum and maximum dates were based on the confidence intervals of 38 previously estimated dates available from TimeTree (Kumar et al., 2017), chosen to capture a broad range of divergences across land plants (**Supplementary Table 3**). This was done in order to constrain our dating analysis to dates that are in line with the general consensus in the literature, as lineage heterogeneity can make results sensitive to taxon sampling when conducting dating analyses with plastome datasets (Beaulieu et al., 2015; Foster et al., 2017). The inferred dated tree and the input for treePL may be found at (https://github.com/alexatyszka/phylorganelles).

The topology for each gene tree was inferred using maximum likelihood as implemented in IQ-TREE v.1.6.12, using the GTR model of evolution with gamma rate heterogeneity. Support for individual edges was inferred with 1,000 UFBoot replicates. The root-to-tip variance for individual gene trees was assessed using the midpoint rooting algorithm implemented in DendroPy (Sukumaran and Holder, 2010), with root-to-tip variance calculated using the program *pxlstr* from the phyx package, using the pipeline (https://github.com/HollyMaeRobertson/RootingAndVariance). Conflict between the COMB-Merged topology and the PLAST-Proportional and MITO-proportional topologies was assessed using a bipartition-based approach (Salichos and Rokas, 2013; Smith et al., 2015) implemented in the program CAnDI (https://github.com/HollyMaeRobertson/gene_family_conflicts).

## Results

### Dataset statistics

Our final dataset consisted of 226 taxa within land plants. Each species was represented by both a sequenced plastome and mitochondrial genome in datasets PLAST and MITO, respectively. Plastid gene occupancy across the dataset ranged from a minimum of 51 genes in *Malania oleifera* to a maximum of 79 genes for 32 species (**Supplementary Figure 1**). Mitochondrial gene occupancy across the dataset was represented by a minimum of nine genes in *Coriandrum sativum* to a maximum of 39 genes within five species (**Supplementary Figure 2**).

### Inferred species relationships and gene properties

**Figure 1:**
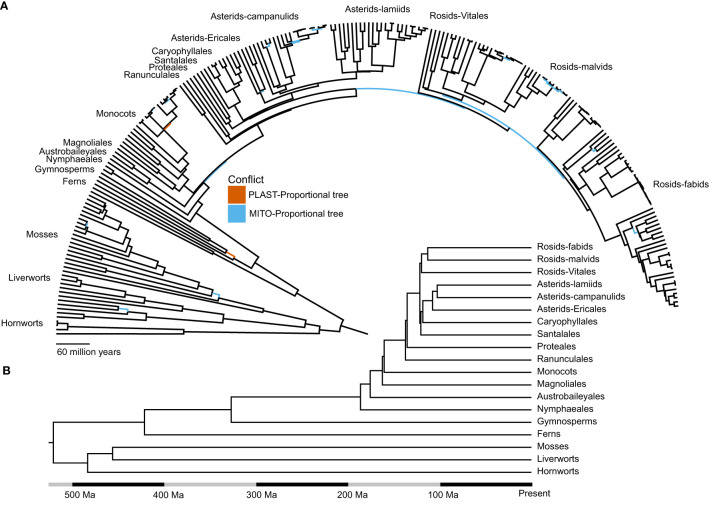
The evolutionary relationships of land plants inferred using organelle data are largely concordant with consensus relationships. **(A)** Species level chronogram for the topology inferred using the COMB-Merged dataset. Conflicts with the PLAST-Proportional topology are highlighted in red, and conflicts with the MITO-Proportional topology are highlighted in blue. **(B)** Chronogram of the COMB-Merged dataset, depicting historically contentious relationships among the major clades in land plants.

Although our sampling did not allow us to test whether bryophytes (hornworts, liverworts and mosses) were monophyletic, all bryophytes formed a single edge in all unrooted phylogenetic trees. Bryophytes, hornworts, liverworts and mosses also each formed single edges in unrooted trees, implying that all of these groups are monophyletic, though lack of sampling from outside land plants precluded our ability to test this. In trees rooted on bryophytes, ferns and gymnosperms were successively sister to angiosperms. Among the angiosperms, we found that in the COMB-Merged topology Nymphaeales, Austrobaileyales, Magnoliales and monocots were successively sister to the eudicots (**Figure 1**). The inferred relationships within these clades predominantly reflected the currently hypothesized relationships from other studies based on nuclear data (e.g., *One Thousand Plant Transcriptomes Initiative, 2019*).

**Figure 2:**
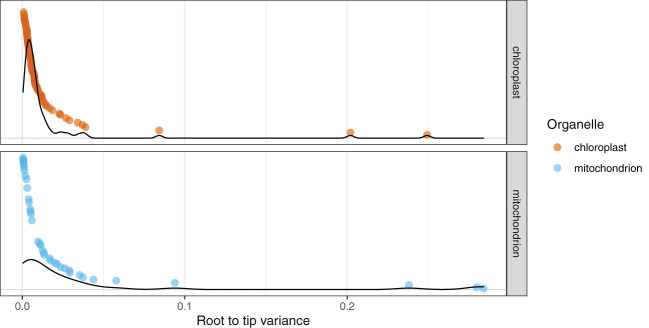
Mitochondrial genes show a broader distribution of root-to-tip variances compared to chloroplast genes. The 79 chloroplast genes are in red and the 39 mitochondrial genes are in blue. Each point is the root-to-tip variance of an individual gene, with the y-axis sorted by root-to-tip variance. Density plots for the respective organelles are overlaid on the graph.

The root-to-tip variance for genes in the PLAST dataset ranged from the highest for *accD* with a value of 0.24914 substitutions/base-pair (subs/bp) to the lowest for *psbE* with a value of 0.00076 (**Figure 2****;** **Supplementary Table 4**). The average value for genes across the PLAST dataset was 0.01477 subs/bp with a median value of 0.00573. The variance for the MITO dataset ranged from *atp9* with a value of 0.28393, to *nad5* with a value of 0.00043 (**Figure 2****;** **Supplementary Table 4**). The average value for the MITO dataset was 0.03506, with a median value of 0.01126.

The PLAST dataset primarily drove the inferred COMB topologies (**Supplementary Table 5**). The overall log-likelihood score for the COMB-Proportional tree was -3268521.55, with -2556885.25 being contributed from the PLAST dataset and -711636.3 being contributed from the MITO dataset.

### Conflict among trees inferred using different datasets

**Figure 3:**
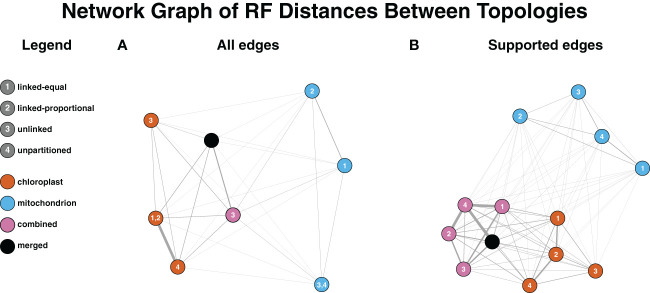
Dataset influences inferred topology more than the partitioning model. The Fruchterman-Reingold algorithm was used to lay out the graph with inverse RF used for edge weights. Nodes are colored based on the dataset and numbered based on the partitioning model. Edge widths on the graph are based on RF distance, with thicker edges corresponding to more similar topologies (smaller RF distances). **(A)** Topologies used for RF distance calculations factored in all edges within datasets regardless of support. Identical topologies from multiple partitioning models result in multiple numbers on the node. For the COMB dataset, all partitioning models aside from edge-unlinked inferred the same topology. **(B)** RF distance calculations are based on topologies where only well-supported edges (≥95% UFBoot) are factored in.

An all-by-all comparison of Robinson-Foulds (RF) distances among the COMB, PLAST and MITO trees demonstrated that differences in topologies were driven more by dataset than partitioning model (**Figure 3**). This pattern arose when the RF values were calculated using all edges, including those lacking strong support (i.e., < 95% UFBoot) (**Figure 3A**; **Supplementary Table 6**), and when only edges with strong support were used (**Figure 3B****;** **Supplementary Table 7**). When the RF analysis was conducted using only well-supported edges, no topologies were found to be identical. Without a support cutoff, topologies inferred with the COMB dataset were identical regardless of the approach, aside from the edge-unlinked model. Regarding trees generated with the PLAST dataset, the PLAST-Equal and PLAST-Proportional topologies were identical. The MITO-Equal and MITO-Unpartitioned topologies were concordant. For RF distances requiring strong support, the two topologies with the greatest distance were those inferred between the COMB-Unlinked and the MITO-Unlinked topologies, which had an RF value of 96. When support was not considered, the COMB-Unlinked topology and the PLAST-Unlinked topology had the largest RF value of 92.

Bipartitions in the COMB-Merged topology were compared to strongly supported (≥ 95% UFBoot) bipartitions in the MITO-Proportional and PLAST-Proportional trees. No nodes of the COMB-Merged topology conflicted with both the PLAST-Proportional and the MITO-Proportional topologies, indicating that all conflicts with this COMB-Merged topology were also conflicts between the PLAST-Proportional and MITO-Proportional topologies.

The PLAST-Proportional topology showed two instances of well-supported conflict with the COMB-Merged topology (**Table 1****;** **Supplementary Figures 3–6**). The first was within the gymnosperm clade, where the PLAST-Proportional placed *Ginkgo biloba* sister to *Cycas taitungensis*. At this node, 11 individual gene trees from the PLAST-Proportional dataset supported the COMB-Merged topology, and 39 supported the PLAST-Proportional topology. Of the individual gene trees with strong support, none of the genes supported the COMB-Merged topology and six genes supported the PLAST-Proportional topology. The other conflict between the PLAST-Proportional topology and the COMB-Merged topology was found at the divergence of *Triticum*, where 23 gene trees supported the COMB-Merged topology and 27 gene trees supported the PLAST-Proportional topology, although no gene tree had strong support for either topology.

**Table 1:** The conflicting relationships between the COMB-Merged and the organelle topologies are reported.

Plastome			
COMB-Merged Relationship	PLAST-Proportional Relationship	PLAST Genes Supporting COMB-Merged (supported)	PLAST Genes Supporting PLAST-Proportional (supported)
*BOP clade*	*Oryza*+*Triticum*	23 (0)	27 (0)
***Pinus*+*Welwitschia+Ginkgo***	***Ginkgo*+*Cycas***	**11 (0)**	**39 (6)**
Mitochondrion			
COMB-Merged Relationship	MITO-Proportional Relationship	MITO Genes Supporting COMB-Merged topology (supported)	MITO Genes Supporting MITO-proportional topology (supported)
*Dumortiera hirsuta*+*Wiesnerella denudata*	*Riccia fluitans*+*Wiesnerella denudata*	8 (0)	9 (0)
*Tetraphis pellucida* sister to other Bryophytina	*Tetraphis pellucida*+*Polytrichaceae*	7 (1)	11 (0)
***Monophyletic Orthotrichum***	***Orthotrichum obtusifolium*+*Stoneobryum bunyaense***	**6 (0)**	**11 (6)**
Monocots+Eudicots	Monocots+Magnoliaceae	0 (0)	4 (1)
*Oryza rufipogon*+*Oryza sativa Indica Group*	*Oryza rufipogon*+*Oryza sativa Japonica Group*	6 (2)	5 (4)
*Chrysopogon zizanioides*+*Saccharum officinarum*+*Sorghum bicolor*	*Chrysopogon zizanioides* sister to other Andropogoneae	13 (1)	4 (1)
*Camellia sinensis*+*Ericaceae*	*Aegiceras corniculatum+Ericaceae*	5 (1)	8 (2)
***Saposhnikovia divaricata*+*Coriandrum sativum***	***Saposhnikovia divaricata*+*Apium graveolens***	**5 (0)**	**23 (21)**
Asteroideae	*Chrysanthemum boreale sister to all other Asteroideae+Lactuca*	4 (0)	6 (0)
*Chrysanthemum boreale*+*Diplostephium hartwegii*	*Chrysanthemum boreale* sister to all other Asteroideae+*Lactuca*	7 (0)	6 (0)
*Bidens pilosa*+*Bidens tripartita*	*Bidens tripartita* sister to all other *Bidens*	0 (0)	15 (1)
*Bidens parviflora*+*Bidens bipinnata*+*Bidens biternata*	*Bidens pilosa+Bidens biternata+Bidens bipinnata*	0 (0)	10 (1)
*Vitis* sister to other Rosids	*Vitis*+core eudicots	2 (0)	3 (0)
Malvids	Rosids missing Vitis sister to *Lagerstroemia indica+Myrtales*	0 (0)	0 (0)
*Gossypium arboreum*+*Gossypium thurberi*	*Gossypium arboreum*+other *Gossypium* aside from *Gossypium barbadense*	3 (3)	5 (0)
*Gossypium arboreum*+*Gossypium thurberi*+*Gossypium hirsutum*+*Gossypium barbadense*	*Gossypium davidsonii* sister to *Gossypium harknessii*+*Gossypium hirsutum*	0 (0)	1 (0)
***Gossypium hirsutum*+*Gossypium barbadense***	***Gossypium barbadense* sister to other *Gossypium***	**0 (0)**	**15 (12)**
*Gossypium raimondii*+*Gossypium trilobum*	*Gossypium trilobum*+*Gossypium thurberi*	0 (0)	2 (0)
*Gossypium harknessii* sister to *Gossypium raimondii*+*Gossypium trilobum*	*Gossypium raimondii* sister to *Gossypium harknessii+Gossypium hirsutum+Gossypium davidsonii*	0 (0)	1 (0)
*Gossypium davidsonii* sister to *Gossypium raimondii+Gossypium trilobum+Gossypium harknessii*	*Gossypium raimondii* sister to *Gossypium davidsonii+Gossypium hirsutum+Gossypium harknessii*	0 (0)	0 (0)
*Raphanus sativus* sister to *Brassica napus+Brassica oleracea+Brassica rapa+Brassica juncea*	*Raphanus sativus* sister to *other Brassiceae*	3 (0)	7 (0)
*Brassica oleracea* sister to *Brassica rapa+Brassica juncea*	*Brassica juncea* sister to *Brassica oleracea+Brassica napus*	14 (13)	0 (0)
*Monophyletic Fabids*	*Euphorbiales* sister to a clade containing *Mangifera longipes+Gossypium harknessii*	2 (0)	5 (0)
*Monophyletic Benincasae*	*Cucumis sativus*+*Cucurbita pepo*	6 (1)	26 (2)
*Gleditsia sinens* sister to other Caesalpinioideae	*Gleditsia sinens* sister to *Senna*	7 (1)	4 (0)

The number of gene trees from the respective organelle concordant with each relationship is reported. The number of gene trees with strong support (≥95% UFBoot) is in parentheses. Bolded relationships are those whose genes are inferred to strongly support the organelle topology.

The MITO-Proportional dataset contained 25 nodes that conflicted with the COMB-Merged dataset (**Table 1****;** **Supplementary Figures 7–10**). Sixteen of these were in divergences inferred to be within the last 25 million years (**Figure 1A**). A focused examination of the support for these conflicts showed that when individual gene tree support is taken into account, only three of the divergences were strongly supported (**Table 1**). These relationships include the monophyly of *Orthotrichum* where six gene trees supported monophyly as inferred in the COMB-Merged topology and 11 genes supported *Orthotrichum obtusifolium* to be sister to *Stoneobryum bunyaense* as inferred in the MITO-Proportional topology. Of the six genes supporting monophyly, none of them were well-supported (≥ 95% UFboot); however, of the 11 gene trees supporting the MITO-Proportional relationship, six of them were well-supported. A similar pattern of bias in conflicting gene tree topologies was found in the early divergences of the cotton genus *Gossypium*, where no gene trees support the COMB-Merged topology and 15 gene trees support the MITO-Proportional topology, with 12 of these gene trees having strong support for the relationship. The other point of conflict was within Apiaceae, where the relationship of *Saposhnikovia divaricata* sister to *Coriandrum sativum* in the COMB-Merged topology is supported by five gene trees and the relationship in the MITO-Proportional topology with *S. divaricata* sister to *Apium graveolens* is supported by 23 gene trees. Of the five gene trees that support the COMB-Merged relationship, none had strong support and 21 of the 23 gene trees that support the MITO-Proportional topology have strong support.

### Combinability of organellar sequences

An assessment of the homogeneity of phylogenetic signal among datasets was conducted by inferring the method that resulted in the best information criterion score and investigating how the individual gene sequences clustered together based on sequence composition and molecular evolution. For the COMB dataset, we found that the PartitionFinder-selected data scheme with a tree inferred using the edge-proportional model showed the best Bayesian Information Criterion (BIC) score (**Table 2**). This is in contrast to the Corrected Akaike Information Criterion (AICc) score, which showed the best score to be the edge-unlinked model. For the plastome dataset (PLAST), the edge-proportional model yielded the best AICc and BIC scores. For the mitochondrion dataset (MITO), the edge-proportional model was selected for BIC and the edge-unlinked model for AICc. The best model for the two organelles was the model in which both evolved under separate topologies.

**Table 2:** Model selection supports the MITO and PLAST datasets as separate topologies. The table is divided into the three datasets whose information criterion values may be compared.

DATASET	IQ-TREE PARTITION MODEL	BIC	AICc	TREE LENGTH
COMB Comparisons				
COMB-Equal	q	6159545.524	6143295.175	11.9499
COMB-Unlinked	sp	6504023.929	6050643.949	12.3044
COMB-Proportional	spp	6130820.047	6113405.119	12.6423
COMB-Unpartitioned	unpartitioned	6204252.327	6199666.787	11.7174
COMB-Merged	MFP+MERGE (spp)	6123413.419	6115155.239	12.5453
MITO-PLAST separate trees	PLAST (spp) + MITO (spp)	**6094588.557**	6072717.621	N/A
MITO-PLAST separate trees	PLAST (spp) + MITO (sp)	620389.145	**6042395.107**	N/A
PLAST Comparisons				
PLAST-Equal	q	4787193.419	4775390.582	13.0565
PLAST-Unlinked	sp	5058998.094	4760900.475	13.2008
PLAST-Proportional	spp	**4772954.87**	**4760411.026**	13.6798
PLAST-Unpartitioned	unpartitioned	4814275.135	4809895.643	12.7412
MITO Comparisons				
MITO-Equal	q	1331030.316	1323526.273	8.78269
MITO-Unlinked	sp	1411663.219	**1288584.971**	11.6034
MITO-Proportional	spp	**1320149.795**	1312307.058	10.4246
MITO-Unpartitioned	unpartitioned	1338539.272	1334427.842	10.3306

The best scoring model by AICc and BIC for each of the three datasets is bolded.

The individual per-gene contribution to the likelihood of the COMB tree demonstrated that the overall contribution to the final tree’s likelihood was largely driven by the genes in the plastome (**Supplementary Table 5**). As inferred using the tree length (sum of all branches in the tree), the plastome showed higher levels of molecular evolution than the mitochondrial genome. This remained true regardless of the partitioning scheme.

**Figure 4:**
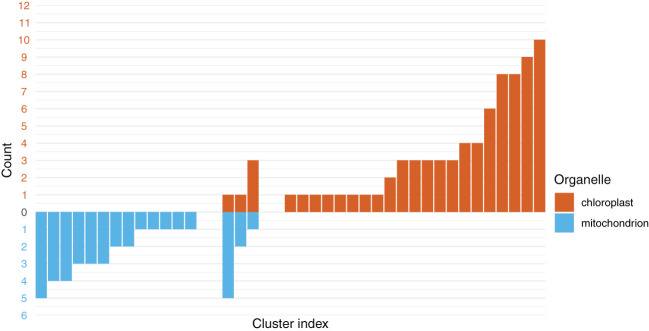
The chloroplast and mitochondrial genes predominantly form clusters with genes from their respective organelles. The y-axis shows the number of genes in a given cluster, with values below 0 and in blue representing the number of mitochondrial genes in a cluster. The values above 0 and in red are the number of chloroplast genes. In the center are the three clusters inferred to contain a mixture of chloroplast and mitochondrial genes.

The PartitionFinder analysis suggested that the best partitioning scheme for the COMB dataset contained 37 model partitions, hereafter, referred to as clusters (**Figure 4****;** **Supplementary Table 8**). Three of the clusters contained a mixture of plastid and mitochondrial genes: one contained five mitochondrial genes and one plastid gene, another contained three plastid genes and one mitochondrial gene, and the final such cluster contained two mitochondrial genes and one plastid gene. Among the homogeneous clusters, which contained genes from only one organellar genome, the largest consisted of 10 plastid genes. Thirteen clusters only contained single genes; of these, five and eight were from the plastome and mitochondrial genomes, respectively.

### Inferred transitions in compositional heterogeneity among major clades

An examination of the compositional heterogeneity shifts showed that when the PLAST and the MITO datasets were analyzed as supermatrices, the PLAST dataset inferred six shifts in compositional heterogeneity while the MITO dataset inferred three (**Supplementary Figures 11, 12**). The three shifts in the MITO dataset were found in the branches subtending bryophytes, seed plants (Spermatophyta), and angiosperms, with the ferns predicted to have retained the ancestral model of evolution. The PLAST dataset also showed compositional shifts at the origins of bryophytes and seed plants as well as at the origin of ferns; the PLAST dataset did not show a shift in heterogeneity at the base of angiosperms but, instead, showed a shift at the divergence of Austrobaileyales and mesangiosperms. Within angiosperms, the PLAST dataset also supported model shifts at the branch subtending monocots + eudicots, as well as at the branches subtending eudicots and core eudicots.

**Figure 5:**
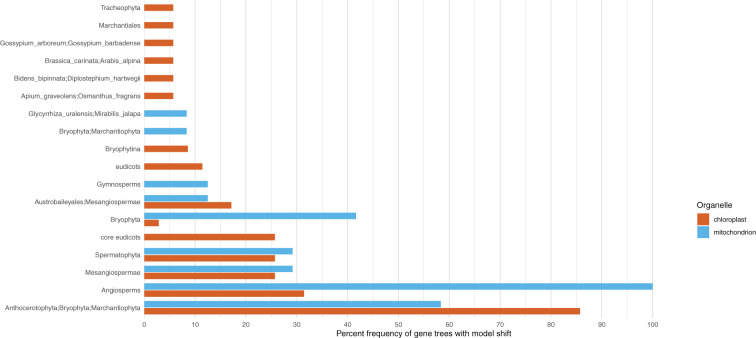
Shifts in compositional heterogeneity are not uniform across or among organelles. Clades with at least five percent of the chloroplast and mitochondrial genes to have an inferred shift in compositional heterogeneity are presented on the y-axis. The x-axis shows the percent of genes with a shift in compositional heterogeneity.

When examining individual genes, we found that the most common compositional shift occurred in the divergence of bryophytes; this was observed in 30 of the 78 PLAST genes (**Figure 5****;** **Supplementary Figure 13****;** **Supplementary Table 9**). The second most common shift occurred at the origin of angiosperms; this was observed in 11 genes. With the exception of the node linking monocots to eudicots, the six most common shifts in compositional heterogeneity for the PLAST dataset genes were divergences found to have inferred shifts for the supermatrix as a whole. When examining the genes of the MITO dataset, we found the most common shift to have occurred at the base of angiosperms, as seen in 24 of the 39 genes (**Figure 5****;** **Supplementary Figure 14****;** **Supplementary Table 10**). With 14 genes, the second most common shift occurred in bryophytes, and the third most common occurred within the bryophytes at the mosses (Bryophyta) with 10 genes. The shift at seed plants seen in the supermatrix dataset was the fourth most common shift, being observed in seven genes.

## Discussion

### Limitations and possible sources of bias as a result of sampling

We limited our sampling to land plants and, therefore, could not evaluate relationships within the bryophytes, including whether the group was monophyletic vs. paraphyletic. Nevertheless, in all unrooted phylogenetic trees, a single bipartition separated bryophytes from vascular plants, and this bipartition was used to root the COMB, PLAST and MITO trees. Our sampling comprised species for which complete sequences of both the plastome and mitochondrial genomes were publicly available. Although our sampling was biased toward angiosperms, with 185 angiosperm species compared to 35 bryophytes, two ferns, and four gymnosperms, this sampling is somewhat proportional to the extant diversity of these major groups (Christenhusz and Byng, 2016). Practically, this means that our evolutionary models will be overwhelmingly informed by sequences from angiosperms. While not ideal, this was unavoidable given the limited number of species with available sequences for both organellar genomes.

Within the ANA grade, we did not include the monotypic order Amborellales in our sampling, despite its evolutionary importance as the sister lineage to all other plants (Soltis et al., 1997; Mathews and Donoghue, 1999; Zanis et al., 2002; *One Thousand Plant Transcriptomes Initiative, 2019*). This was because its mitochondrial genome has a well-documented evolutionary history of horizontal gene transfer from green algae, mosses, and other angiosperms (Rice et al., 2013). Our dataset did include *Welwitschia mirabilis*, which has an increased rate of molecular evolution in its nuclear genomes (De La Torre et al., 2017; Ran et al., 2018) as well as its organellar genomes and a smaller plastome size compared to most land plants (McCoy et al., 2008; Guo et al., 2016). Two other noteworthy species in our sampling were *Viscum album*, known to exhibit a shift in the rate of evolution of the mitochondrial genome due to pseudogenization of several genes (Petersen et al., 2015), and *Geranium maderense*, which is a member of a clade whose mitochondrial genomes have an increased evolutionary rate due to a decrease in RNA editing and transfer of genes from parasitic plants (Park et al., 2015). Indeed, we observed long terminal branch lengths across multiple gene trees leading to the common mistletoe *Viscum album*. Despite the potential for systematic error caused by these exceptional lineages, our phylogenetic methods placed these taxa as expected, based on multiple previous phylogenetic studies (**Figure 1****;** **Supplementary Figures 3–10**). We additionally identified several genes with shifts in molecular rate. Among these was the plastid gene *accD* (acetyl-CoA carboxylase subunit D) in the rice genus *Oryza*; this gene has either been lost or pseudogenized across Poales (Harris et al., 2013). Another gene affected by rate shifts, *rps19*, is duplicated within *Cyperus esculentus* (Ren, 2021) and displayed a shift in molecular rate in our trees. Trimming long branches in gene trees can be used as a strategy to reduce systematic error in phylogenetic analyses. However, due to the difficulty in defining a long-branch trimming cutoff that can accommodate taxa both with shifts in molecular rate that have been documented elsewhere in the literature and those without previously documented shifts in molecular rate, we decided to leave all sequences in the final analyses.

Root-to-tip variance can be used to estimate the extent to which the evolution of genes has been clocklike (Smith et al., 2018) and can be a reliable predictor of phylogenetic accuracy (Vankan et al., 2020). We used root-to-tip variance to estimate biases that may occur due to individual gene tree reconstructions (**Figure 2**). As not all genes contained all outgroups, we used the midpoint rooting method, which assumes clocklike behavior of a gene (Farris, 1972). The majority of genes exhibited low variance (< 0.1 subs/bp), with 36 of the 39 genes in the MITO dataset and 77 of the 79 genes in the PLAST dataset showing this pattern. As a broad trend, mitochondrial genes had higher root-to-tip variances than plastid genes, and in both organellar genomes, the cases in which the variance was > 0.1 subs/bp correlated with factors known to cause rate shifts, such as the *accD* example described above. The lower overall root-to-tip variance across the PLAST dataset indicates that the genes within the plastome evolve in a more clocklike manner.

### Inferred species relationships agree with previous plant systematic studies

Knowledge of the land plant tree of life has been clarified significantly by major recent sequencing efforts, such as the One Thousand Transcriptomes (1Kp) project (Ruhfel et al., 2014; Gitzendanner et al., 2018; *One Thousand Plant Transcriptomes Initiative, 2019*; Yang et al., 2022) and the Plant and Fungal Tree of Life (PAFTOL) project (Baker et al., 2022), as well as by numerous earlier studies employing datasets from Sanger sequencing (e.g., Chase et al., 1993; Chaw et al., 2000; Nickrent et al., 2000; Qiu et al., 2007; Soltis et al., 2011) or earlier methods of plastome sequencing (e.g., Moore et al., 2007; Moore et al., 2010). As sequencing depth continues to increase, obtaining complete plastome and even mitochondrial genomes from short-read data is becoming easier, even when these sequences are not the primary target of study (Weitemier et al., 2014; Morales-Briones et al., 2021). Organellar genome assembly is also being facilitated by advances in methods (e.g., Jin et al., 2020; Wu et al., 2021) and all trends indicate that the plastome and mitochondrial genomes will maintain influential roles in plant phylogenetics.

The results from analyses of both organellar genomes supported topologies that are largely concordant with the current consensus relationships among land plants, which could be attributable to the fact that this consensus has largely been developed from analyses of organellar sequences (Soltis, 2000; Moore et al., 2007). However, as nuclear data have become more prominent, it is becoming common for phylogenetic signals from organellar data to match those of nuclear data, although not without exceptions (e.g., Stull et al., 2020). Most cases of discordance we observed were for historically contentious relationships, such as those among gymnosperms. Overall, both organellar genomes provided similar phylogenetic results for deep and shallow divergences across land plants.

### Optimal partition models support organelles evolving under different trees

When applied to phylogenetics, information criteria provide a statistical framework to identify whether estimated parameters are better modeled under multiple topologies (Theobald, 2010), or whether combining the data and analyzing it under a single topology and set of model parameters provides better information criteria scores (Smith et al., 2020). Information criterion measures can also be used to infer optimal partitioning schemes for multilocus datasets (Lanfear et al., 2012) and how best to account for molecular rate heterogeneity among partitions, which is valuable when some loci have experienced rate shifts (Lopez et al., 2002; Chernomor et al., 2016).

The combinability of data relies upon shared patterns of molecular evolution. The more homogenous the evolutionary processes that underlie the data are, the more combinable the data will be. Information criterion metrics help to infer the number of parameters required to adequately model data. Model parameters are meant to reflect processes of molecular evolution, including processes like rate shifts, changes in substitution rates, and changes in base-pair frequencies. The more combinable sequence data are, the fewer parameters are necessary to model it. Thus, the combinability of data can be a reflection of shared evolutionary history.

Organellar genomes are theoretically uniform in inheritance; despite being composed of separate genes, in the absence of recombination, each should share a single genealogy, and thus represent a single “c-gene” (Doyle, 1992; Doyle, 2022). In practice, however, whether due to biology, analytical error, or a mixture of both, the plastome can appear as a composite of evolutionary histories (Gonçalves et al., 2019; Walker et al., 2019). Similar patterns hold for the mitochondrion (Rokas et al., 2003; Richards et al., 2018). Both organellar genomes have differences in their molecular evolution (Wolfe et al., 1987; Smith and Keeling, 2015), and differences in evolutionary rate across the plastome are significant enough to cause differences in inferred tree topologies (Walker et al., 2014). Therefore, we used partitioning schemes to discern the uniformity of molecular evolution across and among organellar genomes.

Combinability has historically been defined in terms of whether the data support estimating the branch lengths under a single topology as opposed to multiple topologies. Combinability has previously been assessed using Bayes Factors and information criteria (Neupane et al., 2019; Smith et al., 2020). In this study, we examine the combinability of data, both measured in terms of support for multiple topologies, as well as the degree to which the data can be modeled under shared parameters (i.e., the number of parameters contained in the best fit model).

We assessed the degree of combinability with four nested partition models, for which each partition consisted of one aligned gene region. The parameters that contribute to the degree of combinability are the GTR transition matrix (5 parameters), base frequencies (3 parameters), substitution rate variation as measured by invariable sites and a gamma distribution (2 parameters), and edge lengths (2*n*-3 parameters, where *n* is the number of taxa). The least complex model for the data, an unpartitioned model, assumes no heterogeneity in molecular evolution among genes, and therefore does not include separate model partitions for any genes. Increasing in complexity, there is the edge-equal model, where the only parameters shared by the genes are edge lengths. This model allows differences in the sequence’s base frequencies and transition matrices. Therefore, the edge-equal model should have the best fit when shifts in the rate of molecular evolution have not occurred among genes, but changes have occurred at the sequence level. The next partition model we considered, in terms of increasing complexity, is the edge-proportional model (Chernomor et al., 2016), where a speed parameter is included, to accommodate shifts in evolutionary rate (i.e., tree length) across genes, while requiring that the lengths of corresponding branches in all gene trees are proportional to one another. The most complex partition model we considered is the edge-unlinked model (Lopez et al., 2002), where all genes are allowed to vary in evolutionary rate, and rates for a given taxon do not need to be proportional to one another. Modeling two genes with the edge-unlinked model requires the two genes to share a topology, but introduces the same number of parameters as estimating two separate trees with (potentially) different topologies, and thus should always perform worse (or at best, the same) in terms of information criteria than modeling the two with separate trees.

Our results demonstrated that, for all analyses, the edge-proportional model was the best in terms of BIC (**Table 2**). One of our goals was to compare whether it was better to model the evolution of the plastome and mitochondrial genomes separately, or whether a better information criterion score was achieved by modeling the two under shared parameters. When we compared the BIC scores for the combined likelihoods of the separate PLAST- and MITO-inferred topologies to the COMB-inferred topologies, we had to account for the extra branch length parameters introduced by additional edge-proportional model partitions. After accounting for the additional parameters, the results showed that inferring parameters under separate topologies for the plastome and mitochondrial genome provided a better fit to the data (**Table 2**), indicating that in a phylogenetic context, the plastome and mitochondrial genomes are best modeled as separate trees.

To help explain why the histories of plastome and mitochondrial genomes are best modeled as separate trees, despite largely concordant topologies, we examined lineage rate variation and differences in molecular rate. This has been documented to differ significantly between the organelles (Wolfe et al., 1987). We found that the genes within the mitochondrial genome showed a larger discrepancy in root-to-tip variance compared to those of the plastome (**Figure 2**). These differences in root-to-tip variance indicate that the data behaved in a less clock-like manner and, therefore, may be a better fit by a more complex model. We also found that the tree length for the plastome tree was far greater than that of the mitochondrial genome tree (**Table 2**). This difference in rate heterogeneity could explain the lack of combinability, as the separate models can accommodate differences in evolutionary rates between the two organellar genomes by allowing each to have different edge lengths.

When selected based on BIC, the edge-proportional model had the best fit for both the PLAST and MITO datasets (**Table 2**). The edge-proportional model also had the best fit for the PLAST dataset based on AICc. However, AICc supported the edge-unlinked model for the MITO dataset. This discrepancy can be attributed to the lower penalty of AICc compared to BIC for more parameters and that the mitochondrial genes had a broader distribution of root-to-tip variances, indicating greater differences in substitution rate across genes and taxa (**Figure 2**). Both the edge-proportional and edge-unlinked models were developed to accommodate across-partition rate heterogeneity (Lopez et al., 2002; Chernomor et al., 2016). For organellar genomes, our results indicate the importance of incorporating not just heterogeneity in base frequencies or substitution rates but also heterogeneity in rate of molecular evolution among genes, especially as the partition model can influence the inferred topology (**Figure 3**).

Clustering algorithms, such as that implemented in PartitionFinder (Lanfear et al., 2012), identify genes with similar patterns of molecular evolution. We tested whether this algorithm would cluster the PLAST and MITO genes separately, which would indicate that they are evolving under different processes. In these analyses, the 138 genes from the two organelles formed 37 clusters. The inference of multiple clusters reveals heterogeneity across the data. Of these 37 clusters, 34 were homogenous, containing only genes from the PLAST dataset or only genes from the MITO dataset (**Figure 4**). This suggests that genes from the MITO and PLAST datasets largely differed from one another in terms of their patterns of molecular evolution, which may include their substitution rate, base frequency, topology, or some combination of factors which caused them to cluster with other genes from the same organelle. Overall, our results do not support combinability between the organellar genomes, indicating that there are sufficient differences in their molecular evolution to warrant separate trees, likely related to non-topological heterogeneity.

### Topological conflict between organelles and gene tree support

Organelles are often used as textbook examples of uniparental inheritance. However, there are both analytical and biological factors that can cause phylogenies inferred from plastid and mitochondrial data to conflict with one another. This type of phylogenetic conflict has been reported in algae based on analyses of complete plastomes and mitochondrial genomes (Lee et al., 2018). Furthermore, there is evidence of shifts from uniparental to biparental inheritance of organelles across the plant tree of life (Camus et al., 2022). Biparental inheritance may allow for recombination between haplotypes of the same organellar genome (Sullivan et al., 2017; Sancho et al., 2018). Biparental inheritance has been well documented in both mitochondrial genomes (Barr et al., 2005) and plastomes and has arisen independently multiple times (Barnard-Kubow et al., 2017). Biparental inheritance also creates the potential for the two organelles’ evolutionary histories to become unlinked.

The maximum RF distance between any combination of dataset and model, when considering only strongly supported conflict (UFBoot ≥ 95%), was 96. This indicates general topological concordance among the MITO, PLAST, and COMB datasets (**Figure 3B****;** **Supplementary Table 7**), which is to be expected for two genomes whose inheritance patterns are linked. The greatest RF distance was between the COMB-Unlinked and MITO-Unlinked topologies. The edge-unlinked model has the greatest number of parameters, and, therefore, this greater dissimilarity may be explained by there being insufficient data to accurately estimate the large number of model parameters for the unlinked model. The RF distances among trees were influenced more by dataset (MITO, PLAST, and COMB) than by partition model. In addition, trees from the COMB dataset were generally more similar to trees from the PLAST dataset than to those from the MITO dataset (**Figure 3**), which may be due to the larger number of plastid genes and sites constituting the COMB dataset.

An examination of the topological conflicts between the PLAST-Proportional and COMB-Merged uncovered two well-supported conflicting relationships (**Table 1****;** **Supplementary Figures 3, 4**). In comparison, 25 well-supported conflicts were inferred between the COMB-Merged and the MITO-Proportional topologies (**Table 1****;** **Supplementary Figures 7, 8**). This similarity may be explained by the greater number of characters (103,806) in the PLAST supermatrix, compared to the MITO supermatrix (58,295). Furthermore, the individual MITO genes, on average, had a lower contribution to the overall likelihood score of COMB inferred topologies (**Supplementary Table 5**). We observed a slower rate of molecular evolution in the mitochondrial genome compared to the plastome, which has been reported previously (Smith and Keeling, 2015). A slower rate provides fewer informative characters for phylogenetic inference. This same pattern was noted when examining tree length, a proxy for the amount of phylogenetic information, as it is the sum of all branch lengths in the tree. Overall, the plastome appears to be the more informative organellar genome; however, our data can only speak to this with respect to coding sequences. This implies that historical studies which used a combination of mitochondrial and plastid genes in a concatenated supermatrix approach likely recovered the plastome relationship at contentious regions of the tree due to the greater divergence and resulting greater influence of plastid genes (Gatesy and Baker, 2005), although it should be noted this is just one of many factors that might influence phylogenetic inferences (Walker et al., 2020).

When analyzing character-rich supermatrices, bootstrap support may be a misleading metric (Seo, 2008). Therefore, as a second form of support for the PLAST- and MITO-specific trees, we investigated well-supported relationships (UFBoot ≥ 95%) in the gene trees. When applied to data that should share a topology, gene tree concordance provides a conservative subsampling-based support metric. This, complemented with ultrafast bootstrapping, identifies regions of the conflict that warrant further investigation to determine whether the conflict is strictly based on phylogenetic methods or also based on inheritance patterns differing between the organelles. Previous analyses of gene tree support in angiosperms has shown that most conflict between plastome trees and individual gene trees is poorly supported (Walker et al., 2019). Here, we further investigated points of conflict between the COMB-Merged and the PLAST- or MITO-Proportional trees, by identifying how many genes are concordant and well-supported for each topology.

The PLAST-Proportional tree has two points of conflict with the COMB-Merged tree. Therefore, we investigated these further by examining gene tree support. The COMB-Merged topology supported the Bambusoideae-Oryzoideae-Pooideae (“BOP”) clade, whereas the PLAST-Proportional tree placed the genus *Oryza* as sister to the genus *Triticum* (**Table 1****;** **Supplementary Figures 3–6**). There were no gene trees with strong support for either relationship; therefore, we do not consider this to be a supported conflict. The other point of conflict between the PLAST-Proportional and the COMB-Merged topologies was within gymnosperms, where the COMB-Merged tree supported *Cycas* as sister to a clade of *Pinus*, *Welwitschia*, and *Ginkgo*; the PLAST-Proportional tree supported *Ginkgo* as sister to *Cycas*. The relationship within the COMB-Merged topology was supported by 11 gene trees in the COMB dataset; however, none of the gene trees had strong support for the relationship. The PLAST-Proportional topology was supported by 39 gene trees, six of them with strong support for the relationship. The prevalence of biparental inheritance across gymnosperms is unclear, but biparental inheritance of organelles has been documented in *Pinus* (Ni et al., 2021). From a phylogenetic perspective, this conflict in gymnosperms is worthy of further investigation, especially since similar topological conflict has been observed among nuclear gene trees (Stull et al., 2021).

There were 25 points of conflict between the COMB-Merged and the MITO-Proportional trees, and of these, three were strongly supported (≥ 95% UFBoot) in the MITO-Proportional tree (**Table 2****;** **Supplementary Figures 7–10**). Examining the distribution of conflict shows that bryophytes, gymnosperms, and angiosperms all have at least one point of well-supported conflict, indicating that at all major body plan transitions where our sampling allows this to be investigated, at least one relationship conflicts between the plastome and the mitochondrial genome trees. Although evidence for biparental inheritance of organelles in *Gossypium* has, to our knowledge, not been reported, this clade has several reported allopolyploidy events (Chen et al., 2020), which may result in inter-organelle phylogenetic conflict.

### Plant organellar genomes demonstrate a mixture of concordant and discordant shifts in compositional heterogeneity

It is generally appreciated that biological processes can lead to shifts in genomic composition between lineages, resulting in the compositional heterogeneity observed across the tree of life (Foster, 2004). Plastid, mitochondrial, and nuclear genomes all show evidence of shifts in compositional heterogeneity across the phylogeny of land plants (Sousa et al., 2020b; Sousa et al., 2020a; Smith et al., 2022). In some instances, such as the divergence of bryophytes, these shifts may underlie conflicting inferences of evolutionary histories (Cox et al., 2014; Puttick et al., 2018). Here, we investigated whether these shifts in compositional heterogeneity contribute to topological conflict between organellar genomes. We further explored whether the shifts in the organellar genomes are genome-wide or confined to specific genes.

Similar to previous work, we inferred compositional shifts in both the plastome and mitochondrial genome at the edge corresponding to the divergence of bryophytes (Sousa et al., 2020b; Sousa et al., 2020a). In both genomes, the shift occurred in over half of the genes, making it the most common shift found in genes of the plastome and the second most common shift found in genes of the mitochondrial genome (**Figure 5**). For both organellar genomes, ferns were inferred to share the ancestral compositional model, and another shift was inferred at the divergence of seed plants. These points of divergence provide an association of changes in body plan with shifts in compositional heterogeneity. However, the mitochondrial genome exhibited a shift at the base of angiosperms, unlike the plastome. In the mitochondrial genome, all of the genes showed this shift (**Figure 5**). Despite not showing a genome-wide shift, one-third of the plastid genes showed a shift at the base of angiosperms. This indicates that although not genome-wide, many genes appear to have experienced the same shift, and that the mitochondrial and plastid genomes may have experienced similar selective pressures at this point in angiosperm evolutionary history.

Compositional heterogeneity can alter the inferred topology in phylogenetic analyses (Foster, 2004; Puttick et al., 2018), offering an explanation of why conflicting evolutionary histories are sometimes inferred forgenes and organellar genomes whose inheritance should be linked. We found that trees based on the PLAST and MITO datasets conflict at the divergence of magnoliids (here, represented only by Magnoliales), a historically contentious relationship in studies regarding land plants. This conflict correlates with differences in the inferred compositional model (**Supplementary Figures 13, 14**). In the PLAST dataset, magnoliids were inferred to be sister to a clade of monocots and eudicots, with a shift in the model of evolution at the divergence of monocots and eudicots. In the MITO dataset, the magnoliids are sister to monocots, and the two clades share a model of evolution along with eudicots. The correlation between the model and conflict may help explain why this relationship is contentious.

Not all conflicts between the organellar genomes correlate with differences in the inferred model of evolution. The PLAST dataset placed *Macadamia* and *Nelumbo* as sisters to one another, whereas the MITO dataset placed them as a grade, which was sister to the non-Ranunculales eudicots. Despite the conflict, for both the PLAST and MITO datasets, *Macadamia* and *Nelumbo* were predicted to share a model of evolution, indicating that not all conflict may be attributed to compositional shifts.

The gene-wise examination of compositional heterogeneity demonstrated that, in general, individual genes reflected the compositional shifts of the whole genome to which they belong (**Figure 5****;** **Supplementary Figures 13, 14**). As compositional heterogeneity reflects genome evolution, both organellar genomes appear to have undergone significant changes throughout land plant evolution. Further examination of this phenomenon will undoubtedly provide insight into the many pressures that shape organellar genome evolution.

## Conclusions

Over 1 billion years ago, a cyanobacterium, which would eventually become a chloroplast, entered a eukaryotic cell (Collén et al., 2013; Bowles et al., 2022). Since that time, the evolutionary trajectory of the chloroplast has been intertwined with that of the mitochondrion. Although we observed high levels of topological congruence between these organellar genomes, we also identified a few instances of phylogenetic discordance that warrant further investigation. Phylogenetic models continue to support the two genomes as evolving independently, at least from the standpoint of molecular evolution. This is likely due to a combination of differences in the rates of molecular evolution, as well as several independent compositional shifts. This study highlights important aspects of organellar genome evolution, at different points in land plant phylogeny, that are worthy of further exploration as more extensive organellar genomic datasets are generated. More focused sequencing and assembly of mitochondrial genomes (with sampling matched to available plastome sequences) will be important in examining some outstanding questions in greater detail.

## Data availability statement

The datasets presented in this study can be found in online repositories. The names of the repository/repositories and accession number(s) can be found in the article/**Supplementary Material**.

## Author contributions

AT, EB, and JW led the analyses with contributions from M-WG, KR, and HR. All authors contributed to the interpretation of the analyses. AT, EB, DL, GS, and JW led the writing of the manuscript, with input from KR, M-WG, and HR. AT, EB, and DL generated the figures and tables with help from JW. All authors contributed to the article and approved the submitted version.
