# Supplementary material for: Characterizing conflict and congruence of molecular evolution across organellar genome sequences for phylogenetics in land plants
Source: Front Plant Sci. 2023 Mar 30;14:1125107. doi: 10.3389/fpls.2023.1125107 (PMC10098128; doi:10.3389/fpls.2023.1125107)
Supplement: Supplementary file 1 [file DataSheet_1.docx]

Supplementary Material

**Characterizing conflict and congruence of molecular evolution across organellar genome sequences for phylogenetics in land plants**

Alexa S. Tyszka^1^**^*^**, Eric C. Bretz^1^**^*^**, Holly M. Robertson^2^, Miles D. Woodcock-Girard^1^, Karolis Ramanauskas^1^, Drew A. Larson^3^, Gregory W. Stull^4,5^ and *Joseph F. Walker^1^

***Equal Contribution**

**Correspondence:** Joseph F. Walker, [jfw52@uic.edu](mailto:jfw52@uic.edu)

## Supplementary Tables

| Name | TaxID | Mito Accession | Seq Length | Chloro Accession | Seq Length |
| --- | --- | --- | --- | --- | --- |
| Citrus sinensis | 2711 | NC_037463.1 | 640906 | NC_008334.1 | 160129 |
| Dunaliella salina | 3046 | NC_012930.1 | 28331 | NC_016732.1 | 269044 |
| Chlamydomonas reinhardtii | 3055 | NC_001638.1 | 15758 | NC_005353.1 | 203828 |
| Auxenochlorella protothecoides | 3075 | NC_026009.1 | 57274 | NC_023775.1 | 84576 |
| Chlorella sorokiniana | 3076 | NC_024626.1 | 52528 | NC_023835.1 | 109811 |
| Chlorella vulgaris | 3077 | NC_045362.1 | 87477 | NC_001865.1 | 150613 |
| Tetradesmus obliquus | 3088 | NC_002254.1 | 42781 | NC_008101.1 | 161452 |
| Neochloris aquatica | 3099 | NC_024761.1 | 38021 | NC_029670.1 | 166767 |
| Prototheca wickerhamii | 3111 | NC_001613.1 | 55328 | NC_054192.1 | 47997 |
| Ulva prolifera | 3117 | NC_028538.1 | 63845 | NC_036137.1 | 93066 |
| Coleochaete scutata | 3125 | NC_045180.1 | 242024 | NC_030358.1 | 107236 |
| Chlorokybus atmophyticus | 3144 | NC_009630.1 | 201763 | NC_008822.1 | 152254 |
| Pedinomonas minor | 3159 | NC_000892.1 | 25137 | NC_016733.1 | 98340 |
| Scherffelia dubia | 3190 | NC_045363.1 | 78958 | NC_029807.1 | 137161 |
| Polytrichum commune | 3213 | NC_039775.1 | 114831 | NC_060348.1 | 126323 |
| Physcomitrium patens | 3218 | NC_007945.1 | 105340 | NC_037465.1 | 122905 |
| Anthoceros punctatus | 3234 | NC_049003.1 | 228048 | NC_049001.1 | 160692 |
| Psilotum nudum | 3240 | NC_030952.1 | 364070 | NC_003386.1 | 138829 |
| Ginkgo biloba | 3311 | NC_027976.1 | 346544 | NC_016986.1 | 156988 |
| Pinus taeda | 3352 | NC_039746.1 | 1191054 | NC_021440.1 | 121530 |
| Welwitschia mirabilis | 3377 | NC_029130.1 | 978846 | NC_010654.1 | 119726 |
| Liriodendron tulipifera | 3415 | NC_021152.1 | 553721 | NC_008326.1 | 159886 |
| Cannabis sativa | 3483 | NC_029855.1 | 415602 | NC_026562.1 | 153871 |
| Mirabilis jalapa | 3538 | NC_056991.1 | 267334 | NC_041297.1 | 154480 |
| Beta vulgaris subsp. vulgaris | 3555 | NC_002511.2 | 368801 | NC_059012.1 | 149723 |
| Spinacia oleracea | 3562 | NC_035618.1 | 329613 | NC_002202.1 | 150725 |
| Gossypium barbadense | 3634 | NC_028254.1 | 677434 | NC_008641.1 | 160317 |
| Gossypium hirsutum | 3635 | NC_027406.1 | 668584 | NC_007944.1 | 160301 |
| Carica papaya | 3649 | NC_012116.1 | 476890 | NC_010323.1 | 160100 |
| Citrullus lanatus | 3654 | NC_014043.1 | 379236 | NC_032008.1 | 156906 |
| Cucumis sativus | 3659 | NC_016005.1 | 1555935 | NC_007144.1 | 155293 |
| Cucurbita pepo | 3663 | NC_014050.1 | 982833 | NC_038229.1 | 157343 |
| Arabidopsis thaliana | 3702 | NC_037304.1 | 367808 | NC_000932.1 | 154478 |
| Brassica juncea | 3707 | NC_016123.1 | 219766 | NC_028272.1 | 153483 |
| Brassica napus | 3708 | NC_008285.1 | 221853 | NC_016734.1 | 152860 |
| Brassica nigra | 3710 | NC_029182.1 | 232407 | NC_030450.1 | 153633 |
| Brassica rapa | 3711 | NC_049892.1 | 219736 | NC_040849.1 | 153483 |
| Brassica oleracea | 3712 | NC_016118.1 | 360271 | NC_041167.1 | 153364 |
| Raphanus sativus | 3726 | NC_018551.1 | 258426 | NC_024469.1 | 153368 |
| Malus domestica | 3750 | NC_018554.1 | 396947 | NC_061549.1 | 160288 |
| Glycine max | 3847 | NC_020455.1 | 402558 | NC_007942.1 | 152218 |
| Glycine soja | 3848 | NC_039768.1 | 402545 | NC_022868.1 | 152217 |
| Medicago truncatula | 3880 | NC_029641.1 | 271618 | NC_003119.8 | 124033 |
| Phaseolus vulgaris | 3885 | NC_045135.1 | 395516 | NC_009259.1 | 150285 |
| Styphnolobium japonicum | 3897 | NC_039596.1 | 484916 | NC_047059.1 | 158656 |
| Vigna angularis | 3914 | NC_021092.1 | 404466 | NC_021091.1 | 151683 |
| Viscum album | 3972 | NC_029039.1 | 565432 | NC_028012.1 | 128921 |
| Manihot esculenta | 3983 | NC_045136.1 | 682840 | NC_010433.1 | 161453 |
| Ricinus communis | 3988 | NC_015141.1 | 502773 | NC_016736.1 | 163161 |
| Apium graveolens | 4045 | NC_058313.1 | 371275 | NC_041087.1 | 152050 |
| Coriandrum sativum | 4047 | NC_059794.1 | 82926 | NC_029850.1 | 146519 |
| Capsicum annuum | 4072 | NC_024624.1 | 511530 | NC_018552.1 | 156781 |
| Hyoscyamus niger | 4079 | NC_026515.1 | 501401 | NC_024261.1 | 155720 |
| Solanum lycopersicum | 4081 | NC_035963.1 | 446257 | AC_000188.1 | 155461 |
| Nicotiana sylvestris | 4096 | NC_029805.1 | 430597 | NC_007500.1 | 155941 |
| Nicotiana tabacum | 4097 | NC_006581.1 | 430597 | NC_001879.2 | 155943 |
| Solanum melongena | 4111 | NC_050334.1 | 482343 | NC_030207.1 | 154289 |
| Solanum tuberosum | 4113 | NC_059127.1 | 112834 | NC_008096.2 | 155296 |
| Arctium lappa | 4217 | NC_058644.1 | 312598 | NC_042724.1 | 152708 |
| Helianthus annuus | 4232 | NC_023337.1 | 300945 | NC_007977.1 | 151104 |
| Helianthus tuberosus | 4233 | NC_058585.1 | 281287 | NC_023112.1 | 151047 |
| Lactuca sativa | 4236 | NC_042756.1 | 363324 | NC_007578.1 | 152765 |
| Nelumbo nucifera | 4432 | NC_030753.1 | 524797 | NC_025339.1 | 163330 |
| Camellia sinensis | 4442 | NC_043914.1 | 707441 | NC_020019.1 | 157103 |
| Oryza rufipogon | 4529 | NC_013816.1 | 559045 | NC_017835.1 | 134544 |
| Saccharum officinarum | 4547 | NC_031164.1 | 300784 | NC_035224.1 | 141176 |
| Sorghum bicolor | 4558 | NC_008360.1 | 468628 | NC_008602.1 | 140754 |
| Tripsacum dactyloides | 4563 | NC_008362.1 | 704100 | NC_037087.1 | 141050 |
| Triticum aestivum | 4565 | NC_036024.1 | 452526 | NC_002762.1 | 134545 |
| Triticum timopheevii | 4570 | NC_022714.1 | 443419 | NC_024764.1 | 136157 |
| Zea perennis | 4580 | NC_008331.1 | 570354 | NC_030300.1 | 140647 |
| Allium cepa | 4679 | NC_030100.1 | 316363 | NC_024813.1 | 153538 |
| Asparagus officinalis | 4686 | NC_053642.1 | 492062 | NC_034777.1 | 156699 |
| Schisandra sphenanthera | 13674 | NC_042758.1 | 1101768 | NC_037145.1 | 146843 |
| Tetracentron sinense | 13715 | NC_059859.1 | 856059 | NC_021425.1 | 164467 |
| Vaccinium macrocarpon | 13750 | NC_023338.1 | 459678 | NC_019616.1 | 176045 |
| Sphagnum palustre | 13805 | NC_024521.1 | 141276 | NC_030198.1 | 140040 |
| Nothoceros aenigmaticus | 13813 | NC_012651.1 | 184908 | NC_020259.1 | 153208 |
| Cocos nucifera | 13894 | NC_031696.1 | 678653 | NC_022417.1 | 154731 |
| Zea luxurians | 15945 | NC_008333.1 | 539368 | NC_030301.1 | 140710 |
| Solanum pennellii | 28526 | NC_035964.1 | 423596 | NC_035742.1 | 155254 |
| Fagus sylvatica | 28930 | NC_050960.1 | 504715 | NC_041437.1 | 158462 |
| Geranium maderense | 28964 | NC_027000.1 | 737091 | NC_029999.1 | 155694 |
| Funaria hygrometrica | 29583 | NC_024523.1 | 109586 | NC_058544.1 | 122213 |
| Zostera marina | 29655 | NC_035345.1 | 191481 | NC_036014.1 | 143877 |
| Spirodela polyrhiza | 29656 | NC_017840.1 | 228493 | NC_015891.1 | 168788 |
| Eleusine indica | 29674 | NC_040989.1 | 520691 | NC_030486.1 | 135151 |
| Sinapis arvensis | 29728 | NC_031896.1 | 240024 | NC_035303.1 | 153590 |
| Gossypium arboreum | 29729 | NC_035073.1 | 687482 | NC_016712.1 | 160230 |
| Gossypium raimondii | 29730 | NC_029998.1 | 676078 | NC_016668.1 | 160161 |
| Vitis vinifera | 29760 | NC_012119.1 | 773279 | NC_007957.1 | 160928 |
| Mychonastes homosphaera | 31300 | NC_024760.1 | 25149 | NC_029671.1 | 102718 |
| Chromochloris zofingiensis | 31302 | NC_024758.1 | 44840 | NC_029672.1 | 188935 |
| Nephroselmis olivacea | 31312 | NC_008239.1 | 45223 | NC_000927.1 | 200799 |
| Gonium pectorale | 33097 | NC_020437.1 | 15993 | NC_020438.1 | 222582 |
| Gossypium thurberi | 34273 | NC_035074.1 | 644395 | NC_015204.1 | 160264 |
| Gossypium trilobum | 34281 | NC_035076.1 | 644460 | NC_033397.1 | 160109 |
| Gossypium harknessii | 34285 | NC_027407.1 | 666081 | NC_033333.1 | 160129 |
| Gossypium davidsonii | 34287 | NC_035075.1 | 644311 | NC_033395.1 | 160072 |
| Lotus japonicus | 34305 | NC_016743.2 | 380861 | NC_002694.1 | 150519 |
| Ipomoea nil | 35883 | NC_031158.1 | 265768 | NC_031159.1 | 161897 |
| Sorbus aucuparia | 36599 | NC_052880.1 | 384977 | NC_052878.1 | 160108 |
| Cymbomonas tetramitiformis | 36881 | NC_036614.1 | 73520 | NC_030169.1 | 84524 |
| Pyramimonas parkeae | 36894 | NC_031504.1 | 43294 | NC_012099.1 | 101605 |
| Atrichum angustatum | 37310 | NC_024520.1 | 115146 | NC_058541.1 | 125602 |
| Citrus maxima | 37334 | NC_057143.1 | 538434 | NC_034290.1 | 160133 |
| Tetraphis pellucida | 37420 | NC_024290.1 | 107730 | NC_024291.1 | 127489 |
| Silene latifolia | 37657 | NC_014487.1 | 253413 | NC_016730.1 | 151736 |
| Ajuga reptans | 38596 | NC_023103.1 | 352069 | NC_023102.1 | 149963 |
| Botryococcus braunii | 38881 | NC_027722.1 | 84583 | NC_025545.1 | 172826 |
| Aneura pinguis | 39026 | NC_026901.1 | 165603 | NC_035617.1 | 120698 |
| Dracocephalum moldavica | 39296 | NC_062584.1 | 150177 | NC_057509.1 | 149868 |
| Agrostemma githago | 39848 | NC_057604.1 | 262903 | NC_023357.1 | 151733 |
| Oryza sativa Indica Group | 39946 | NC_007886.1 | 491515 | NC_008155.1 | 134496 |
| Oryza sativa Japonica Group | 39947 | NC_011033.1 | 490520 | NC_001320.1 | 134525 |
| Anthoceros agrestis | 41834 | NC_049004.1 | 228021 | NC_049002.1 | 160790 |
| Riccia fluitans | 41844 | NC_043906.1 | 185621 | NC_042887.1 | 121682 |
| Bathycoccus prasinos | 41875 | NC_023273.1 | 43614 | NC_024811.1 | 72700 |
| Pycnococcus provasolii | 41880 | NC_013935.1 | 24321 | NC_012097.1 | 80211 |
| Mesostigma viride | 41882 | NC_008240.1 | 42424 | NC_002186.1 | 118360 |
| Prunus avium | 42229 | NC_044768.1 | 389709 | NC_044701.1 | 157886 |
| Bidens pilosa | 42337 | NC_062673.1 | 183061 | NC_046793.1 | 150542 |
| Phoenix dactylifera | 42345 | NC_016740.1 | 715001 | NC_013991.2 | 158462 |
| Populus alba | 43335 | NC_041085.1 | 838420 | NC_008235.1 | 156505 |
| Roya anglica | 43943 | NC_046950.1 | 69371 | NC_024168.1 | 138275 |
| Haematococcus lacustris | 44745 | NC_044670.1 | 124604 | NC_037007.1 | 1352306 |
| Bombax ceiba | 45325 | NC_038052.1 | 594390 | NC_037494.1 | 158997 |
| Bupleurum falcatum | 46367 | NC_035962.1 | 463792 | NC_027834.1 | 155989 |
| Anthoceros angustus | 48387 | NC_037476.1 | 242410 | NC_004543.1 | 161162 |
| Asclepias syriaca | 48545 | NC_022796.1 | 682498 | NC_022432.1 | 158719 |
| Nicotiana attenuata | 49451 | NC_036467.1 | 394341 | NC_035952.1 | 155886 |
| Callicladium imponens | 49762 | NC_024516.1 | 103830 | NC_058545.1 | 125195 |
| Bracteacoccus minor | 50037 | NC_024756.1 | 45175 | NC_029674.1 | 192761 |
| Bracteacoccus aerius | 50041 | NC_024755.1 | 47158 | NC_029675.1 | 165732 |
| Butomus umbellatus | 50236 | NC_021399.1 | 450826 | NC_051949.1 | 158107 |
| Arabis alpina | 50452 | NC_037070.1 | 323159 | NC_023367.1 | 152866 |
| Pisum fulvum | 51020 | NC_059792.1 | 379922 | NC_036828.1 | 120837 |
| Bidens tripartita | 51276 | NC_062671.1 | 216786 | NC_058915.1 | 150489 |
| Oltmannsiellopsis viridis | 51324 | NC_008256.1 | 56761 | NC_008099.1 | 151933 |
| Yamagishiella unicocca | 51707 | NC_033969.1 | 30876 | NC_039754.1 | 300175 |
| Brassica carinata | 52824 | NC_016120.1 | 232241 | NC_059807.1 | 153641 |
| Bartramia pomiformis | 52976 | NC_024519.1 | 106198 | NC_050047.1 | 125886 |
| Cycas taitungensis | 54799 | NC_010303.1 | 414903 | NC_009618.1 | 163403 |
| Chara vulgaris | 55564 | NC_005255.1 | 67737 | NC_008097.1 | 184933 |
| Pongamia pinnata | 56065 | NC_016742.1 | 425718 | NC_016708.2 | 152968 |
| Marchantia paleacea | 56867 | NC_001660.1 | 186609 | NC_001319.1 | 121024 |
| Dumortiera hirsuta | 56917 | NC_042873.1 | 178019 | NC_039590.1 | 122050 |
| Trifolium pratense | 57577 | NC_048499.1 | 301823 | NC_047412.1 | 146573 |
| Sapindus mukorossi | 57655 | NC_050850.1 | 602121 | NC_025554.1 | 160481 |
| Tamarindus indica | 58860 | NC_045038.1 | 607282 | NC_026685.1 | 159551 |
| Aegiceras corniculatum | 59970 | NC_056358.1 | 425282 | NC_045111.1 | 157241 |
| Fragaria nubicola | 60188 | NC_062587.1 | 299474 | NC_058807.1 | 155608 |
| Macadamia integrifolia | 60698 | NC_058888.1 | 682814 | NC_025288.1 | 159714 |
| Orthotrichum obtusifolium | 61564 | NC_031767.1 | 104603 | NC_026979.1 | 122895 |
| Ulva linza | 63409 | NC_029701.1 | 70858 | NC_030312.1 | 86726 |
| Chenopodium quinoa | 63459 | NC_041093.1 | 315003 | NC_034949.1 | 152099 |
| Oryza minuta | 63629 | NC_029816.1 | 515022 | NC_030298.1 | 135094 |
| Ulva compressa | 63659 | NC_041082.1 | 62477 | NC_050739.1 | 96808 |
| Fragaria iinumae | 64939 | NC_062591.1 | 328819 | NC_024258.1 | 155554 |
| Fragaria moschata | 64940 | NC_062590.1 | 277672 | NC_062593.1 | 155601 |
| Fragaria nilgerrensis | 64941 | NC_062589.1 | 315211 | NC_058806.1 | 155783 |
| Fragaria viridis | 64942 | NC_062592.1 | 289289 | NC_048474.1 | 155458 |
| Gleditsia sinensis | 66096 | NC_058235.1 | 594121 | NC_047282.1 | 163175 |
| Buxbaumia aphylla | 70128 | NC_024518.1 | 100725 | NC_046056.1 | 123907 |
| Climacium dendroides | 70131 | NC_053886.1 | 104860 | NC_051864.1 | 124957 |
| Ostreococcus tauri | 70448 | NC_008290.1 | 44237 | NC_008289.1 | 71666 |
| Eucalyptus grandis | 71139 | NC_040010.1 | 478813 | NC_014570.1 | 160137 |
| Boechera stricta | 72658 | NC_042143.1 | 271601 | NC_049599.1 | 155033 |
| Helianthus grosseserratus | 73291 | NC_051989.1 | 273543 | NC_023108.1 | 151017 |
| Glycyrrhiza uralensis | 74613 | NC_053919.1 | 463869 | NC_047343.1 | 127887 |
| Ulva rigida | 75689 | NC_053633.1 | 88416 | NC_053616.1 | 118206 |
| Salix purpurea | 77065 | NC_029693.1 | 598970 | NC_026722.1 | 155590 |
| Passiflora edulis | 78168 | NC_050950.1 | 680480 | NC_034285.1 | 151406 |
| Spondias mombin | 80338 | NC_045035.1 | 674158 | NC_035973.1 | 162302 |
| Populus tremula x Populus alba | 80863 | NC_028329.1 | 783513 | NC_028504.1 | 156641 |
| Cuscuta japonica | 81913 | NC_060804.1 | 813731 | NC_060789.1 | 120975 |
| Capsella rubella | 81985 | NC_042883.1 | 287405 | NC_027693.1 | 154601 |
| Ulva flexuosa | 83791 | NC_035809.1 | 71545 | NC_035823.1 | 89414 |
| Ulva fenestrata | 83795 | NC_053629.1 | 59026 | NC_053612.1 | 94654 |
| Magnolia officinalis | 85864 | NC_064401.1 | 930306 | NC_020316.1 | 160183 |
| Magnolia biondii | 86725 | NC_049134.1 | 967100 | NC_034687.1 | 160002 |
| Scyphiphora hydrophyllacea | 86994 | NC_057654.1 | 354155 | NC_049078.1 | 155132 |
| Picocystis salinarum | 88271 | NC_042491.1 | 41858 | NC_024828.1 | 81133 |
| Pectinodesmus pectinatus | 91197 | NC_036659.1 | 32195 | NC_036668.1 | 196809 |
| Corchorus olitorius | 93759 | NC_031360.1 | 1829341 | NC_044468.1 | 161766 |
| Osmanthus fragrans | 93977 | NC_060346.1 | 563202 | NC_042377.1 | 155896 |
| Platycodon grandiflorus | 94286 | NC_035958.1 | 1249593 | NC_035624.1 | 171818 |
| Chaetosphaeridium globosum | 96477 | NC_004118.1 | 56574 | NC_004115.1 | 131183 |
| Trifolium grandiflorum | 97024 | NC_048501.1 | 347723 | NC_024034.1 | 125628 |
| Fragaria gracilis | 101011 | NC_062834.1 | 283031 | NC_062837.1 | 155630 |
| Fragaria orientalis | 101013 | NC_057524.1 | 275143 | NC_035501.1 | 147835 |
| Roya obtusa | 104537 | NC_022863.1 | 69465 | NC_030315.1 | 138272 |
| Ulva australis | 111616 | NC_053628.1 | 64466 | NC_053611.1 | 109820 |
| Ulva fasciata | 111617 | NC_028081.1 | 61614 | NC_029040.1 | 96005 |
| Ammopiptanthus nanus | 111851 | NC_046466.1 | 339352 | NC_034743.1 | 154140 |
| Populus tremula | 113636 | NC_028096.1 | 783442 | NC_027425.1 | 156067 |
| Rhododendron simsii | 118357 | NC_053763.1 | 802707 | NC_053764.1 | 152214 |
| Ostreobium quekettii | 121088 | NC_045361.1 | 241739 | NC_030629.1 | 81997 |
| Hesperelaea palmeri | 126429 | NC_031323.1 | 658522 | NC_025787.1 | 155820 |
| Senna occidentalis | 126820 | NC_038221.1 | 447106 | NC_038222.1 | 159993 |
| Ammopiptanthus mongolicus | 126911 | NC_039660.1 | 475396 | NC_034742.1 | 153935 |
| Entransia fimbriata | 130991 | NC_022861.1 | 61645 | NC_030313.1 | 206025 |
| Triosteum pinnatifidum | 134526 | NC_064333.1 | 803609 | NC_037952.1 | 154896 |
| Calystegia soldanella | 136204 | NC_060803.1 | 279265 | NC_060788.1 | 152317 |
| Acacia ligulata | 138025 | NC_040998.1 | 698138 | NC_026134.2 | 174233 |
| Sanionia uncinata | 140003 | NC_027974.1 | 104497 | NC_025668.1 | 124374 |
| Pohlia nutans | 140635 | NC_046778.1 | 99864 | NC_045869.1 | 125199 |
| Lagerstroemia indica | 141186 | NC_035616.1 | 333948 | NC_030484.1 | 152205 |
| Monomastix sp. OKE-1 | 141716 | NC_022797.1 | 60883 | NC_012101.1 | 114528 |
| Monoraphidium neglectum | 145388 | NW_014013625.1 | 93840 | NW_014013626.1 | 135362 |
| Helicosporidium sp. ex Simulium jonesi | 145475 | NC_017841.1 | 49343 | NC_008100.1 | 37454 |
| Bougainvillea spectabilis | 146096 | NC_056281.1 | 343746 | NC_041266.1 | 154541 |
| Caulerpa lentillifera | 148947 | NC_038217.1 | 209034 | NC_039377.1 | 119402 |
| Prasinoderma coloniale | 156133 | NC_023355.1 | 54546 | NC_024817.1 | 77750 |
| Cynanchum auriculatum | 157409 | NC_041494.1 | 426495 | NC_029460.1 | 160840 |
| Vigna radiata | 157791 | NC_015121.1 | 401262 | NC_013843.1 | 151271 |
| Tupiella akineta | 160070 | NC_005926.1 | 95880 | NC_008114.1 | 195867 |
| Chlorotetraedron incus | 162317 | NC_024757.1 | 38406 | NC_029673.1 | 193197 |
| Chrysopogon zizanioides | 167337 | NC_056367.1 | 551622 | NC_035034.1 | 139971 |
| Caulerpa ashmeadii | 177078 | NC_045849.1 | 197427 | NC_045914.1 | 135722 |
| Wiesnerella denudata | 179049 | NC_053538.1 | 186911 | NC_050394.1 | 122500 |
| Ilex pubescens | 185543 | NC_045078.1 | 517520 | NC_057154.1 | 157872 |
| Myurella julacea | 186697 | NC_054351.1 | 104979 | NC_053275.1 | 124457 |
| Leucaena trichandra | 190760 | NC_039738.1 | 722009 | NC_028733.1 | 164692 |
| Haematoxylum brasiletto | 191923 | NC_045040.1 | 631094 | NC_026679.1 | 157728 |
| Utricularia reniformis | 192314 | NC_034982.1 | 857234 | NC_029719.2 | 139725 |
| Pisum abyssinicum | 198035 | NC_059791.1 | 354692 | NC_037830.1 | 122174 |
| Saposhnikovia divaricata | 203717 | NC_058846.1 | 293897 | NC_050292.1 | 147834 |
| Solanum aethiopicum | 205524 | NC_050335.1 | 566695 | NC_039608.1 | 155608 |
| Gymnomitrion concinnatum | 209793 | NC_040132.1 | 162572 | NC_040133.1 | 120994 |
| Douinia plicata | 209816 | NC_054214.1 | 144205 | NC_051003.1 | 118797 |
| Corchorus capsularis | 210143 | NC_031359.1 | 1999602 | NC_044467.1 | 161088 |
| Nymphaea colorata | 210225 | NC_037468.1 | 617195 | NC_057562.1 | 159842 |
| Aquilaria sinensis | 210372 | NC_054354.1 | 341829 | NC_029243.1 | 159565 |
| Salvia miltiorrhiza | 226208 | NC_023209.1 | 499236 | NC_020431.1 | 151328 |
| Hibiscus cannabinus | 229543 | NC_035549.1 | 569915 | NC_045873.1 | 162903 |
| Aconitum kusnezoffii | 239685 | NC_053920.1 | 440720 | NC_031422.1 | 155862 |
| Ptychomnion cygnisetum | 245469 | NC_024514.1 | 104480 | NC_058547.1 | 123452 |
| Diplophyllum taxifolium | 248355 | NC_054215.1 | 144129 | NC_053890.1 | 118309 |
| Leiosporoceros dussii | 263836 | NC_039751.1 | 212153 | NC_039750.1 | 155956 |
| Populus davidiana | 266767 | NC_035157.1 | 779361 | NC_032717.1 | 155853 |
| Ulota hutchinsiae | 279761 | NC_024517.1 | 104608 | NC_058548.1 | 123615 |
| Nowellia curvifolia | 280839 | NC_063925.1 | 148199 | NC_063926.1 | 114423 |
| Micromonas commoda | 296587 | NC_012643.1 | 47425 | NC_012575.1 | 72585 |
| Lobosphaera incisa | 312850 | NC_027060.1 | 69997 | NC_025533.1 | 156031 |
| Libidibia coriaria | 321550 | NC_045039.1 | 601574 | NC_026677.1 | 158045 |
| Ziziphus jujuba | 326968 | NC_029809.1 | 365190 | NC_030299.1 | 161466 |
| Pleodorina starrii | 330485 | NC_021108.1 | 20375 | NC_021109.1 | 269857 |
| Chlorosarcinopsis eremi | 332213 | NC_041430.1 | 24905 | NC_042250.1 | 298847 |
| Beta macrocarpa | 343494 | NC_015994.1 | 385220 | NC_059016.1 | 149727 |
| Chrysanthemum boreale | 344871 | NC_039757.1 | 211002 | NC_037388.1 | 151012 |
| Beta vulgaris subsp. maritima | 350892 | NC_015099.1 | 364950 | NC_059015.1 | 149724 |
| Melicope pteleifolia | 354501 | NC_050882.1 | 159012 | NC_053871.1 | 159014 |
| Trifolium aureum | 361561 | NC_048502.1 | 294911 | NC_024035.1 | 126970 |
| Trifolium meduseum | 361940 | NC_048500.1 | 348724 | NC_024166.1 | 142595 |
| Senna tora | 362788 | NC_038053.1 | 566589 | NC_030193.1 | 162426 |
| Helianthus strumosus | 382522 | NC_051990.1 | 281056 | NC_023113.1 | 151044 |
| Hepatica maxima | 387291 | NC_053368.1 | 1122546 | NC_045909.1 | 160876 |
| Rhazya stricta | 396313 | NC_024293.1 | 548608 | NC_024292.1 | 154841 |
| Suaeda glauca | 397272 | NC_060419.1 | 474330 | NC_045303.1 | 149807 |
| Malania oleifera | 397392 | NC_053625.1 | 527575 | NC_039426.1 | 125050 |
| Evolvulus alsinoides | 439689 | NC_058741.1 | 344184 | NC_058590.1 | 157015 |
| Physochlaina orientalis | 451527 | NC_044153.1 | 684857 | NC_044154.1 | 156321 |
| Chloropicon laureae | 464258 | NC_042492.1 | 37769 | NC_042484.1 | 64740 |
| Bidens biternata | 468846 | NC_062672.1 | 198476 | NC_060634.1 | 151487 |
| Dorcoceras hygrometricum | 472368 | NC_016741.1 | 510519 | NC_016468.1 | 153493 |
| Mirabilis himalaica | 482968 | NC_048974.1 | 346363 | NC_048975.1 | 154348 |
| Orthotrichum stellatum | 522327 | NC_024522.1 | 104131 | NC_058546.1 | 123253 |
| Ulva gigantea | 523338 | NC_053630.1 | 66743 | NC_053613.1 | 117606 |
| Fragaria tibetica | 538578 | NC_062832.1 | 283001 | NC_062835.1 | 155643 |
| Fragaria iturupensis | 538579 | NC_062833.1 | 351183 | NC_062836.1 | 155637 |
| Prunus salicina x Prunus armeniaca | 551137 | NC_060491.1 | 484858 | NC_060490.1 | 157916 |
| Chlorella variabilis | 554065 | NC_025413.1 | 78500 | NC_015359.1 | 124579 |
| Coccomyxa subellipsoidea C-169 | 574566 | NC_015316.1 | 65497 | NC_015084.1 | 175731 |
| Nymphaea hybrid cultivar | 637490 | NC_060361.1 | 335042 | NC_060360.1 | 159968 |
| Pseudomuriella schumacherensis | 889459 | NC_024763.1 | 43134 | NC_029669.1 | 220357 |
| Morus notabilis | 981085 | NC_041177.1 | 362069 | NC_027110.1 | 158680 |
| Spondias tuberosa | 991123 | NC_045036.1 | 779106 | NC_030527.1 | 162039 |
| Jenufa perforata | 993091 | NC_046779.1 | 27198 | NC_028581.1 | 198040 |
| Jenufa minuta | 993092 | NC_046780.1 | 41488 | NC_028582.1 | 206680 |
| Acer yangbiense | 1000413 | NC_059858.1 | 803281 | NC_050351.1 | 155706 |
| Tolypanthus maclurei | 1026954 | NC_056836.1 | 256961 | NC_042257.1 | 123581 |
| Chlamydomonas leiostraca | 1034604 | NC_026573.1 | 14029 | NC_032109.1 | 167394 |
| Cyperus esculentus | 1053340 | NC_058697.1 | 1002696 | NC_058698.1 | 186255 |
| Scapania ampliata | 1133302 | NC_052751.1 | 143664 | NC_051002.1 | 118026 |
| Trebouxiophyceae sp. MX-AZ01 | 1208065 | NC_018568.1 | 74423 | NC_018569.1 | 149707 |
| Ophioglossum californicum | 1267209 | NC_030900.1 | 372339 | NC_020147.1 | 138270 |
| Salix suchowensis | 1278906 | NC_029317.1 | 644437 | NC_026462.1 | 155214 |
| Chloroparvula japonica | 1411623 | NC_042601.1 | 40432 | NC_042487.1 | 71262 |
| Salix dunnii | 1413687 | NC_058734.1 | 711422 | NC_058985.1 | 155647 |
| Closterium baillyanum | 1416941 | NC_022860.1 | 152089 | NC_030314.1 | 201341 |
| Chloropicon maureeniae | 1461542 | NC_042602.1 | 36041 | NC_042488.1 | 64094 |
| Chloropicon roscoffensis | 1461544 | NC_042599.1 | 38736 | NC_042486.1 | 64534 |
| Marchantia polymorpha subsp. ruderalis | 1480154 | NC_037508.1 | 186196 | NC_037507.1 | 120304 |
| Bidens parviflora | 1527830 | NC_062670.1 | 195825 | NC_060633.1 | 151314 |
| Bidens bipinnata | 1527831 | NC_060635.1 | 198476 | NC_062669.1 | 151486 |
| Epirixanthes elongata | 1562316 | NC_046014.1 | 365168 | NC_046013.1 | 36275 |
| Chloropicon mariensis | 1606511 | NC_042600.1 | 35545 | NC_042485.1 | 64323 |
| Syntrichia filaris | 1678074 | NC_027515.1 | 106343 | NC_050352.1 | 136227 |
| Diplostephium hartwegii | 1716017 | NC_034354.1 | 277718 | NC_034832.1 | 151994 |
| Nepenthes ventricosa x Nepenthes alata | 1744888 | NC_039531.1 | 520764 | NC_044185.1 | 156637 |
| Chloropicon sieburthii | 1764286 | NC_042598.1 | 37591 | NC_042483.1 | 64565 |
| Castilleja paramensis | 1857654 | NC_031806.1 | 495499 | NC_031805.1 | 152926 |
| Chloroparvula pacifica | 1883388 | NC_042603.1 | 49744 | NC_042489.1 | 69644 |
| Stoneobryum bunyaense | 1903712 | NC_031392.1 | 104352 | NC_042479.1 | 123040 |
| Prototheca ciferrii | 1973153 | NC_037449.1 | 38164 | NC_037450.1 | 28698 |
| Salix brachista | 2182728 | NC_058733.1 | 608983 | NC_058984.1 | 155600 |
| Prototheca bovis | 2509265 | NC_045058.1 | 39222 | NC_045059.1 | 28638 |
| Mangifera longipes | 2762383 | NC_060990.1 | 728635 | NC_057291.1 | 157853 |
| Pseudanomodon attenuatus | 2779804 | NC_021931.1 | 104252 | NC_058540.1 | 125282 |
| Ulva lacinulata | 2806530 | NC_053631.1 | 79723 | NC_053614.1 | 103444 |
| Rhaphiolepis bibas | 2843217 | NC_045228.1 | 434980 | NC_034639.1 | 159137 |
| Ulva sp. A AF-2021 | 2847172 | NC_053632.1 | 88318 | NC_053615.1 | 96673 |
| Leontynka pallida | 2912034 | NC_063674.1 | 104812 | NC_063675.1 | 362307 |
| Torminalis glaberrima | 2914000 | NC_052879.1 | 386758 | NC_033975.1 | 160390 |

**Supplementary table 1.** Initial 316 taxa dataset of all green plant species on genbank that contain a complete and annotated plastome and mitochondrial genome.

| Name | TaxID | Mito Accession | Seq Length | Chloro Accession | Seq Length |
| --- | --- | --- | --- | --- | --- |
| Citrus sinensis | 2711 | NC_037463.1 | 640906 | NC_008334.1 | 160129 |
| Polytrichum commune | 3213 | NC_039775.1 | 114831 | NC_060348.1 | 126323 |
| Physcomitrium patens | 3218 | NC_007945.1 | 105340 | NC_037465.1 | 122905 |
| Anthoceros punctatus | 3234 | NC_049003.1 | 228048 | NC_049001.1 | 160692 |
| Psilotum nudum | 3240 | NC_030952.1 | 364070 | NC_003386.1 | 138829 |
| Ginkgo biloba | 3311 | NC_027976.1 | 346544 | NC_016986.1 | 156988 |
| Pinus taeda | 3352 | NC_039746.1 | 1191054 | NC_021440.1 | 121530 |
| Welwitschia mirabilis | 3377 | NC_029130.1 | 978846 | NC_010654.1 | 119726 |
| Liriodendron tulipifera | 3415 | NC_021152.1 | 553721 | NC_008326.1 | 159886 |
| Cannabis sativa | 3483 | NC_029855.1 | 415602 | NC_026562.1 | 153871 |
| Mirabilis jalapa | 3538 | NC_056991.1 | 267334 | NC_041297.1 | 154480 |
| Spinacia oleracea | 3562 | NC_035618.1 | 329613 | NC_002202.1 | 150725 |
| Gossypium barbadense | 3634 | NC_028254.1 | 677434 | NC_008641.1 | 160317 |
| Gossypium hirsutum | 3635 | NC_027406.1 | 668584 | NC_007944.1 | 160301 |
| Carica papaya | 3649 | NC_012116.1 | 476890 | NC_010323.1 | 160100 |
| Citrullus lanatus | 3654 | NC_014043.1 | 379236 | NC_032008.1 | 156906 |
| Cucumis sativus | 3659 | NC_016005.1 | 1555935 | NC_007144.1 | 155293 |
| Cucurbita pepo | 3663 | NC_014050.1 | 982833 | NC_038229.1 | 157343 |
| Arabidopsis thaliana | 3702 | NC_037304.1 | 367808 | NC_000932.1 | 154478 |
| Brassica juncea | 3707 | NC_016123.1 | 219766 | NC_028272.1 | 153483 |
| Brassica napus | 3708 | NC_008285.1 | 221853 | NC_016734.1 | 152860 |
| Brassica nigra | 3710 | NC_029182.1 | 232407 | NC_030450.1 | 153633 |
| Brassica rapa | 3711 | NC_049892.1 | 219736 | NC_040849.1 | 153483 |
| Brassica oleracea | 3712 | NC_016118.1 | 360271 | NC_041167.1 | 153364 |
| Raphanus sativus | 3726 | NC_018551.1 | 258426 | NC_024469.1 | 153368 |
| Malus domestica | 3750 | NC_018554.1 | 396947 | NC_061549.1 | 160288 |
| Glycine max | 3847 | NC_020455.1 | 402558 | NC_007942.1 | 152218 |
| Glycine soja | 3848 | NC_039768.1 | 402545 | NC_022868.1 | 152217 |
| Medicago truncatula | 3880 | NC_029641.1 | 271618 | NC_003119.8 | 124033 |
| Phaseolus vulgaris | 3885 | NC_045135.1 | 395516 | NC_009259.1 | 150285 |
| Styphnolobium japonicum | 3897 | NC_039596.1 | 484916 | NC_047059.1 | 158656 |
| Vigna angularis | 3914 | NC_021092.1 | 404466 | NC_021091.1 | 151683 |
| Viscum album | 3972 | NC_029039.1 | 565432 | NC_028012.1 | 128921 |
| Manihot esculenta | 3983 | NC_045136.1 | 682840 | NC_010433.1 | 161453 |
| Ricinus communis | 3988 | NC_015141.1 | 502773 | NC_016736.1 | 163161 |
| Apium graveolens | 4045 | NC_058313.1 | 371275 | NC_041087.1 | 152050 |
| Coriandrum sativum | 4047 | NC_059794.1 | 82926 | NC_029850.1 | 146519 |
| Capsicum annuum | 4072 | NC_024624.1 | 511530 | NC_018552.1 | 156781 |
| Hyoscyamus niger | 4079 | NC_026515.1 | 501401 | NC_024261.1 | 155720 |
| Solanum lycopersicum | 4081 | NC_035963.1 | 446257 | AC_000188.1 | 155461 |
| Nicotiana sylvestris | 4096 | NC_029805.1 | 430597 | NC_007500.1 | 155941 |
| Nicotiana tabacum | 4097 | NC_006581.1 | 430597 | NC_001879.2 | 155943 |
| Solanum melongena | 4111 | NC_050334.1 | 482343 | NC_030207.1 | 154289 |
| Solanum tuberosum | 4113 | NC_059127.1 | 112834 | NC_008096.2 | 155296 |
| Arctium lappa | 4217 | NC_058644.1 | 312598 | NC_042724.1 | 152708 |
| Helianthus annuus | 4232 | NC_023337.1 | 300945 | NC_007977.1 | 151104 |
| Helianthus tuberosus | 4233 | NC_058585.1 | 281287 | NC_023112.1 | 151047 |
| Lactuca sativa | 4236 | NC_042756.1 | 363324 | NC_007578.1 | 152765 |
| Nelumbo nucifera | 4432 | NC_030753.1 | 524797 | NC_025339.1 | 163330 |
| Camellia sinensis | 4442 | NC_043914.1 | 707441 | NC_020019.1 | 157103 |
| Oryza rufipogon | 4529 | NC_013816.1 | 559045 | NC_017835.1 | 134544 |
| Saccharum officinarum | 4547 | NC_031164.1 | 300784 | NC_035224.1 | 141176 |
| Sorghum bicolor | 4558 | NC_008360.1 | 468628 | NC_008602.1 | 140754 |
| Tripsacum dactyloides | 4563 | NC_008362.1 | 704100 | NC_037087.1 | 141050 |
| Triticum aestivum | 4565 | NC_036024.1 | 452526 | NC_002762.1 | 134545 |
| Triticum timopheevii | 4570 | NC_022714.1 | 443419 | NC_024764.1 | 136157 |
| Zea perennis | 4580 | NC_008331.1 | 570354 | NC_030300.1 | 140647 |
| Allium cepa | 4679 | NC_030100.1 | 316363 | NC_024813.1 | 153538 |
| Asparagus officinalis | 4686 | NC_053642.1 | 492062 | NC_034777.1 | 156699 |
| Schisandra sphenanthera | 13674 | NC_042758.1 | 1101768 | NC_037145.1 | 146843 |
| Vaccinium macrocarpon | 13750 | NC_023338.1 | 459678 | NC_019616.1 | 176045 |
| Sphagnum palustre | 13805 | NC_024521.1 | 141276 | NC_030198.1 | 140040 |
| Cocos nucifera | 13894 | NC_031696.1 | 678653 | NC_022417.1 | 154731 |
| Zea luxurians | 15945 | NC_008333.1 | 539368 | NC_030301.1 | 140710 |
| Solanum pennellii | 28526 | NC_035964.1 | 423596 | NC_035742.1 | 155254 |
| Fagus sylvatica | 28930 | NC_050960.1 | 504715 | NC_041437.1 | 158462 |
| Geranium maderense | 28964 | NC_027000.1 | 737091 | NC_029999.1 | 155694 |
| Funaria hygrometrica | 29583 | NC_024523.1 | 109586 | NC_058544.1 | 122213 |
| Zostera marina | 29655 | NC_035345.1 | 191481 | NC_036014.1 | 143877 |
| Spirodela polyrhiza | 29656 | NC_017840.1 | 228493 | NC_015891.1 | 168788 |
| Eleusine indica | 29674 | NC_040989.1 | 520691 | NC_030486.1 | 135151 |
| Sinapis arvensis | 29728 | NC_031896.1 | 240024 | NC_035303.1 | 153590 |
| Gossypium arboreum | 29729 | NC_035073.1 | 687482 | NC_016712.1 | 160230 |
| Gossypium raimondii | 29730 | NC_029998.1 | 676078 | NC_016668.1 | 160161 |
| Vitis vinifera | 29760 | NC_012119.1 | 773279 | NC_007957.1 | 160928 |
| Gossypium thurberi | 34273 | NC_035074.1 | 644395 | NC_015204.1 | 160264 |
| Gossypium trilobum | 34281 | NC_035076.1 | 644460 | NC_033397.1 | 160109 |
| Gossypium harknessii | 34285 | NC_027407.1 | 666081 | NC_033333.1 | 160129 |
| Gossypium davidsonii | 34287 | NC_035075.1 | 644311 | NC_033395.1 | 160072 |
| Lotus japonicus | 34305 | NC_016743.2 | 380861 | NC_002694.1 | 150519 |
| Ipomoea nil | 35883 | NC_031158.1 | 265768 | NC_031159.1 | 161897 |
| Sorbus aucuparia | 36599 | NC_052880.1 | 384977 | NC_052878.1 | 160108 |
| Atrichum angustatum | 37310 | NC_024520.1 | 115146 | NC_058541.1 | 125602 |
| Citrus maxima | 37334 | NC_057143.1 | 538434 | NC_034290.1 | 160133 |
| Tetraphis pellucida | 37420 | NC_024290.1 | 107730 | NC_024291.1 | 127489 |
| Silene latifolia | 37657 | NC_014487.1 | 253413 | NC_016730.1 | 151736 |
| Ajuga reptans | 38596 | NC_023103.1 | 352069 | NC_023102.1 | 149963 |
| Aneura pinguis | 39026 | NC_026901.1 | 165603 | NC_035617.1 | 120698 |
| Agrostemma githago | 39848 | NC_057604.1 | 262903 | NC_023357.1 | 151733 |
| Oryza sativa Indica Group | 39946 | NC_007886.1 | 491515 | NC_008155.1 | 134496 |
| Oryza sativa Japonica Group | 39947 | NC_011033.1 | 490520 | NC_001320.1 | 134525 |
| Anthoceros agrestis | 41834 | NC_049004.1 | 228021 | NC_049002.1 | 160790 |
| Riccia fluitans | 41844 | NC_043906.1 | 185621 | NC_042887.1 | 121682 |
| Prunus avium | 42229 | NC_044768.1 | 389709 | NC_044701.1 | 157886 |
| Bidens pilosa | 42337 | NC_062673.1 | 183061 | NC_046793.1 | 150542 |
| Phoenix dactylifera | 42345 | NC_016740.1 | 715001 | NC_013991.2 | 158462 |
| Populus alba | 43335 | NC_041085.1 | 838420 | NC_008235.1 | 156505 |
| Bombax ceiba | 45325 | NC_038052.1 | 594390 | NC_037494.1 | 158997 |
| Anthoceros angustus | 48387 | NC_037476.1 | 242410 | NC_004543.1 | 161162 |
| Asclepias syriaca | 48545 | NC_022796.1 | 682498 | NC_022432.1 | 158719 |
| Nicotiana attenuata | 49451 | NC_036467.1 | 394341 | NC_035952.1 | 155886 |
| Callicladium imponens | 49762 | NC_024516.1 | 103830 | NC_058545.1 | 125195 |
| Butomus umbellatus | 50236 | NC_021399.1 | 450826 | NC_051949.1 | 158107 |
| Arabis alpina | 50452 | NC_037070.1 | 323159 | NC_023367.1 | 152866 |
| Pisum fulvum | 51020 | NC_059792.1 | 379922 | NC_036828.1 | 120837 |
| Bidens tripartita | 51276 | NC_062671.1 | 216786 | NC_058915.1 | 150489 |
| Brassica carinata | 52824 | NC_016120.1 | 232241 | NC_059807.1 | 153641 |
| Bartramia pomiformis | 52976 | NC_024519.1 | 106198 | NC_050047.1 | 125886 |
| Cycas taitungensis | 54799 | NC_010303.1 | 414903 | NC_009618.1 | 163403 |
| Pongamia pinnata | 56065 | NC_016742.1 | 425718 | NC_016708.2 | 152968 |
| Marchantia paleacea | 56867 | NC_001660.1 | 186609 | NC_001319.1 | 121024 |
| Dumortiera hirsuta | 56917 | NC_042873.1 | 178019 | NC_039590.1 | 122050 |
| Trifolium pratense | 57577 | NC_048499.1 | 301823 | NC_047412.1 | 146573 |
| Sapindus mukorossi | 57655 | NC_050850.1 | 602121 | NC_025554.1 | 160481 |
| Tamarindus indica | 58860 | NC_045038.1 | 607282 | NC_026685.1 | 159551 |
| Aegiceras corniculatum | 59970 | NC_056358.1 | 425282 | NC_045111.1 | 157241 |
| Fragaria nubicola | 60188 | NC_062587.1 | 299474 | NC_058807.1 | 155608 |
| Macadamia integrifolia | 60698 | NC_058888.1 | 682814 | NC_025288.1 | 159714 |
| Orthotrichum obtusifolium | 61564 | NC_031767.1 | 104603 | NC_026979.1 | 122895 |
| Chenopodium quinoa | 63459 | NC_041093.1 | 315003 | NC_034949.1 | 152099 |
| Oryza minuta | 63629 | NC_029816.1 | 515022 | NC_030298.1 | 135094 |
| Fragaria iinumae | 64939 | NC_062591.1 | 328819 | NC_024258.1 | 155554 |
| Fragaria moschata | 64940 | NC_062590.1 | 277672 | NC_062593.1 | 155601 |
| Fragaria nilgerrensis | 64941 | NC_062589.1 | 315211 | NC_058806.1 | 155783 |
| Fragaria viridis | 64942 | NC_062592.1 | 289289 | NC_048474.1 | 155458 |
| Gleditsia sinensis | 66096 | NC_058235.1 | 594121 | NC_047282.1 | 163175 |
| Buxbaumia aphylla | 70128 | NC_024518.1 | 100725 | NC_046056.1 | 123907 |
| Climacium dendroides | 70131 | NC_053886.1 | 104860 | NC_051864.1 | 124957 |
| Eucalyptus grandis | 71139 | NC_040010.1 | 478813 | NC_014570.1 | 160137 |
| Boechera stricta | 72658 | NC_042143.1 | 271601 | NC_049599.1 | 155033 |
| Helianthus grosseserratus | 73291 | NC_051989.1 | 273543 | NC_023108.1 | 151017 |
| Glycyrrhiza uralensis | 74613 | NC_053919.1 | 463869 | NC_047343.1 | 127887 |
| Salix purpurea | 77065 | NC_029693.1 | 598970 | NC_026722.1 | 155590 |
| Passiflora edulis | 78168 | NC_050950.1 | 680480 | NC_034285.1 | 151406 |
| Spondias mombin | 80338 | NC_045035.1 | 674158 | NC_035973.1 | 162302 |
| Populus tremula x Populus alba | 80863 | NC_028329.1 | 783513 | NC_028504.1 | 156641 |
| Cuscuta japonica | 81913 | NC_060804.1 | 813731 | NC_060789.1 | 120975 |
| Capsella rubella | 81985 | NC_042883.1 | 287405 | NC_027693.1 | 154601 |
| Magnolia officinalis | 85864 | NC_064401.1 | 930306 | NC_020316.1 | 160183 |
| Magnolia biondii | 86725 | NC_049134.1 | 967100 | NC_034687.1 | 160002 |
| Scyphiphora hydrophyllacea | 86994 | NC_057654.1 | 354155 | NC_049078.1 | 155132 |
| Corchorus olitorius | 93759 | NC_031360.1 | 1829341 | NC_044468.1 | 161766 |
| Osmanthus fragrans | 93977 | NC_060346.1 | 563202 | NC_042377.1 | 155896 |
| Platycodon grandiflorus | 94286 | NC_035958.1 | 1249593 | NC_035624.1 | 171818 |
| Trifolium grandiflorum | 97024 | NC_048501.1 | 347723 | NC_024034.1 | 125628 |
| Fragaria gracilis | 101011 | NC_062834.1 | 283031 | NC_062837.1 | 155630 |
| Fragaria orientalis | 101013 | NC_057524.1 | 275143 | NC_035501.1 | 147835 |
| Ammopiptanthus nanus | 111851 | NC_046466.1 | 339352 | NC_034743.1 | 154140 |
| Populus tremula | 113636 | NC_028096.1 | 783442 | NC_027425.1 | 156067 |
| Rhododendron simsii | 118357 | NC_053763.1 | 802707 | NC_053764.1 | 152214 |
| Hesperelaea palmeri | 126429 | NC_031323.1 | 658522 | NC_025787.1 | 155820 |
| Senna occidentalis | 126820 | NC_038221.1 | 447106 | NC_038222.1 | 159993 |
| Triosteum pinnatifidum | 134526 | NC_064333.1 | 803609 | NC_037952.1 | 154896 |
| Calystegia soldanella | 136204 | NC_060803.1 | 279265 | NC_060788.1 | 152317 |
| Acacia ligulata | 138025 | NC_040998.1 | 698138 | NC_026134.2 | 174233 |
| Sanionia uncinata | 140003 | NC_027974.1 | 104497 | NC_025668.1 | 124374 |
| Pohlia nutans | 140635 | NC_046778.1 | 99864 | NC_045869.1 | 125199 |
| Lagerstroemia indica | 141186 | NC_035616.1 | 333948 | NC_030484.1 | 152205 |
| Bougainvillea spectabilis | 146096 | NC_056281.1 | 343746 | NC_041266.1 | 154541 |
| Cynanchum auriculatum | 157409 | NC_041494.1 | 426495 | NC_029460.1 | 160840 |
| Vigna radiata | 157791 | NC_015121.1 | 401262 | NC_013843.1 | 151271 |
| Chrysopogon zizanioides | 167337 | NC_056367.1 | 551622 | NC_035034.1 | 139971 |
| Wiesnerella denudata | 179049 | NC_053538.1 | 186911 | NC_050394.1 | 122500 |
| Ilex pubescens | 185543 | NC_045078.1 | 517520 | NC_057154.1 | 157872 |
| Myurella julacea | 186697 | NC_054351.1 | 104979 | NC_053275.1 | 124457 |
| Leucaena trichandra | 190760 | NC_039738.1 | 722009 | NC_028733.1 | 164692 |
| Haematoxylum brasiletto | 191923 | NC_045040.1 | 631094 | NC_026679.1 | 157728 |
| Pisum abyssinicum | 198035 | NC_059791.1 | 354692 | NC_037830.1 | 122174 |
| Saposhnikovia divaricata | 203717 | NC_058846.1 | 293897 | NC_050292.1 | 147834 |
| Solanum aethiopicum | 205524 | NC_050335.1 | 566695 | NC_039608.1 | 155608 |
| Gymnomitrion concinnatum | 209793 | NC_040132.1 | 162572 | NC_040133.1 | 120994 |
| Douinia plicata | 209816 | NC_054214.1 | 144205 | NC_051003.1 | 118797 |
| Corchorus capsularis | 210143 | NC_031359.1 | 1999602 | NC_044467.1 | 161088 |
| Nymphaea colorata | 210225 | NC_037468.1 | 617195 | NC_057562.1 | 159842 |
| Aquilaria sinensis | 210372 | NC_054354.1 | 341829 | NC_029243.1 | 159565 |
| Hibiscus cannabinus | 229543 | NC_035549.1 | 569915 | NC_045873.1 | 162903 |
| Aconitum kusnezoffii | 239685 | NC_053920.1 | 440720 | NC_031422.1 | 155862 |
| Ptychomnion cygnisetum | 245469 | NC_024514.1 | 104480 | NC_058547.1 | 123452 |
| Diplophyllum taxifolium | 248355 | NC_054215.1 | 144129 | NC_053890.1 | 118309 |
| Leiosporoceros dussii | 263836 | NC_039751.1 | 212153 | NC_039750.1 | 155956 |
| Populus davidiana | 266767 | NC_035157.1 | 779361 | NC_032717.1 | 155853 |
| Ulota hutchinsiae | 279761 | NC_024517.1 | 104608 | NC_058548.1 | 123615 |
| Nowellia curvifolia | 280839 | NC_063925.1 | 148199 | NC_063926.1 | 114423 |
| Libidibia coriaria | 321550 | NC_045039.1 | 601574 | NC_026677.1 | 158045 |
| Ziziphus jujuba | 326968 | NC_029809.1 | 365190 | NC_030299.1 | 161466 |
| Chrysanthemum boreale | 344871 | NC_039757.1 | 211002 | NC_037388.1 | 151012 |
| Trifolium aureum | 361561 | NC_048502.1 | 294911 | NC_024035.1 | 126970 |
| Trifolium meduseum | 361940 | NC_048500.1 | 348724 | NC_024166.1 | 142595 |
| Senna tora | 362788 | NC_038053.1 | 566589 | NC_030193.1 | 162426 |
| Helianthus strumosus | 382522 | NC_051990.1 | 281056 | NC_023113.1 | 151044 |
| Hepatica maxima | 387291 | NC_053368.1 | 1122546 | NC_045909.1 | 160876 |
| Rhazya stricta | 396313 | NC_024293.1 | 548608 | NC_024292.1 | 154841 |
| Suaeda glauca | 397272 | NC_060419.1 | 474330 | NC_045303.1 | 149807 |
| Malania oleifera | 397392 | NC_053625.1 | 527575 | NC_039426.1 | 125050 |
| Evolvulus alsinoides | 439689 | NC_058741.1 | 344184 | NC_058590.1 | 157015 |
| Physochlaina orientalis | 451527 | NC_044153.1 | 684857 | NC_044154.1 | 156321 |
| Bidens biternata | 468846 | NC_062672.1 | 198476 | NC_060634.1 | 151487 |
| Dorcoceras hygrometricum | 472368 | NC_016741.1 | 510519 | NC_016468.1 | 153493 |
| Mirabilis himalaica | 482968 | NC_048974.1 | 346363 | NC_048975.1 | 154348 |
| Orthotrichum stellatum | 522327 | NC_024522.1 | 104131 | NC_058546.1 | 123253 |
| Fragaria tibetica | 538578 | NC_062832.1 | 283001 | NC_062835.1 | 155643 |
| Fragaria iturupensis | 538579 | NC_062833.1 | 351183 | NC_062836.1 | 155637 |
| Prunus salicina x Prunus armeniaca | 551137 | NC_060491.1 | 484858 | NC_060490.1 | 157916 |
| Nymphaea hybrid cultivar | 637490 | NC_060361.1 | 335042 | NC_060360.1 | 159968 |
| Morus notabilis | 981085 | NC_041177.1 | 362069 | NC_027110.1 | 158680 |
| Spondias tuberosa | 991123 | NC_045036.1 | 779106 | NC_030527.1 | 162039 |
| Acer yangbiense | 1000413 | NC_059858.1 | 803281 | NC_050351.1 | 155706 |
| Tolypanthus maclurei | 1026954 | NC_056836.1 | 256961 | NC_042257.1 | 123581 |
| Cyperus esculentus | 1053340 | NC_058697.1 | 1002696 | NC_058698.1 | 186255 |
| Scapania ampliata | 1133302 | NC_052751.1 | 143664 | NC_051002.1 | 118026 |
| Ophioglossum californicum | 1267209 | NC_030900.1 | 372339 | NC_020147.1 | 138270 |
| Salix suchowensis | 1278906 | NC_029317.1 | 644437 | NC_026462.1 | 155214 |
| Salix dunnii | 1413687 | NC_058734.1 | 711422 | NC_058985.1 | 155647 |
| Marchantia polymorpha subsp. ruderalis | 1480154 | NC_037508.1 | 186196 | NC_037507.1 | 120304 |
| Bidens parviflora | 1527830 | NC_062670.1 | 195825 | NC_060633.1 | 151314 |
| Bidens bipinnata | 1527831 | NC_060635.1 | 198476 | NC_062669.1 | 151486 |
| Syntrichia filaris | 1678074 | NC_027515.1 | 106343 | NC_050352.1 | 136227 |
| Diplostephium hartwegii | 1716017 | NC_034354.1 | 277718 | NC_034832.1 | 151994 |
| Nepenthes ventricosa x Nepenthes alata | 1744888 | NC_039531.1 | 520764 | NC_044185.1 | 156637 |
| Castilleja paramensis | 1857654 | NC_031806.1 | 495499 | NC_031805.1 | 152926 |
| Stoneobryum bunyaense | 1903712 | NC_031392.1 | 104352 | NC_042479.1 | 123040 |
| Salix brachista | 2182728 | NC_058733.1 | 608983 | NC_058984.1 | 155600 |
| Mangifera longipes | 2762383 | NC_060990.1 | 728635 | NC_057291.1 | 157853 |
| Pseudanomodon attenuatus | 2779804 | NC_021931.1 | 104252 | NC_058540.1 | 125282 |
| Rhaphiolepis bibas | 2843217 | NC_045228.1 | 434980 | NC_034639.1 | 159137 |
| Torminalis glaberrima | 2914000 | NC_052879.1 | 386758 | NC_033975.1 | 160390 |

**Supplementary table 2.** Refined 226 taxa dataset of all land plant species used in this study.

| **Genus 1** | **Genus 2** | **DT (MYA)** | **CI (MYA)** |  |  |  |  |
| --- | --- | --- | --- | --- | --- | --- | --- |
| Trifolium | Vigna | 55 | 50.4 - 74.0 |  |  | **DT: Divergence Time** |  |
| Vigna | Acacia | 60 | 19.6 - 66.9 |  |  | **CI: Confidence Interval** |  |
| Acacia | Fragaria | 99 | 89.2 - 104.5 |  |  |  |  |
| Fragaria | Rhaphiolepis | 59 | 44.5 - 89.3 |  |  | Eudicots | |
| Rhaphiolepis | Cannabis | 87 | 73.6 - 90.2 |  |  | Monocots | |
| Cannabis | Cucumis | 101 | 89.0 - 105.9 |  |  | Magnoliids | |
| Cucumis | Salix | 101 | 99.0 - 110.8 |  |  | Tracheophytes | |
| Salix | Brassica | 108 | 102.0 - 113.8 |  |  | Pinophyta | |
| Brassica | Gossypium | 90 | 83.0 - 93.1 |  |  | Anthocerotophyta | |
| Gossypium | Citrus | 95 | 90.0 - 99.9 |  |  | Bryophyta | |
| Citrus | Solanum | 120 | 112.4 - 125.0 |  |  | Marchantiophyta | |
| Solanum | Cuscuta | 60 | 56.2 - 83.9 |  |  | Polypodiophyta | |
| Cuscuta | Cynanchum | 77 | 72.4 - 104.9 |  |  |  | |
| Cynanchum | Bidens | 101 | 97.5 - 107.3 |  |  |  |  |
| Bidens | Apium | 77 | 75.6 - 90.4 |  |  |  |  |
| Apium | Rhododendron | 108 | 104.2 - 115.6 |  |  |  |  |
| Rhododendron | Spinacia | 116 | 110.3 - 122.0 |  |  |  |  |
| Spinacia | Viscum | 119 | 106.8 - 132.0 |  |  |  |  |
| Viscum | Aconitum | 129 | 126.0 - 136.9 |  |  |  |  |
| Aconitum | Saccharum | 160 | 143.0 - 174.8 |  |  |  |  |
| Saccharum | Oryza | 47 | 40.3 - 51.9 |  |  |  |  |
| Oryza | Cyperus | 94 | 77.7 - 113.0 |  |  |  |  |
| Cyperus | Asparagus | 117 | 108.4 - 122.0 |  |  |  |  |
| Asparagus | Magnolia | 160 | 151.6 - 164.6 |  |  |  |  |
| Magnolia | Nymphaea | 179 | 168.4 - 198.1 |  |  |  |  |
| Nymphaea | Pinus | 330 | 326.4 - 336.8 |  |  |  |  |
| Pinus | Anthoceros | 460 | 440.0 - 524.1 |  |  |  |  |
| Anthoceros | Callicladium | 488 | 452.0 - 632.0 |  |  |  |  |
| Callicladium | Orthotrichum | 80 | 71.7 - 105.1 |  |  |  |  |
| Orthotrichum | Pohlia | 195 | **NA** |  |  |  |  |
| Pohlia | Buxbaumia | 206 | 187.2 - 291.0 |  |  |  |  |
| Buxbaumia | Sphagnum | 306 | 236.9 - 380.4 |  |  |  |  |
| Sphagnum | Scapania | 480 | 455.3 - 510.9 |  |  |  |  |
| Scapania | Aneura | 336 | 228.3 - 361.0 |  |  |  |  |
| Aneura | Wiesnerella | 372 | 304.8 - 437.9 |  |  |  |  |
| Wiesnerella | Riccia | 163 | **NA** |  |  |  |  |
| Riccia | Ophioglossum | 480 | 455.3 - 510.9 |  |  |  |  |
| Polypodiophyta | Welwitschia | 405 | 398.0 - 420.7 |  |  |  |  |

**Supplementary table 3.** Divergences used to help calibrate the chronogram. The inferred divergence time and the confidence intervals from TimeTree.org.

| gene | root_tip_var |
| --- | --- |
| cp__psbt.aln.fasta.treefile | 0.00999784 |
| cp__ccsa.aln.fasta.treefile | 0.00734685 |
| cp__ndhf.aln.fasta.treefile | 0.0113128 |
| cp__rps14.aln.fasta.treefile | 0.00279251 |
| cp__rpl32.aln.fasta.treefile | 0.084209 |
| cp__ycf3.aln.fasta.treefile | 0.00153772 |
| cp__ycf2.aln.fasta.treefile | 0.00769462 |
| cp__rps11.aln.fasta.treefile | 0.00695941 |
| cp__petn.aln.fasta.treefile | 0.00122775 |
| cp__ycf4.aln.fasta.treefile | 0.0168021 |
| cp__atpa.aln.fasta.treefile | 0.0040079 |
| cp__ndhc.aln.fasta.treefile | 0.0119518 |
| cp__psbk.aln.fasta.treefile | 0.00428485 |
| cp__psba.aln.fasta.treefile | 0.00189434 |
| cp__rpob.aln.fasta.treefile | 0.00307738 |
| cp__petg.aln.fasta.treefile | 0.00565348 |
| cp__rps12.aln.fasta.treefile | 0.00332787 |
| cp__rps2.aln.fasta.treefile | 0.00356883 |
| cp__psbj.aln.fasta.treefile | 0.00828897 |
| cp__accd.aln.fasta.treefile | 0.249147 |
| cp__psbf.aln.fasta.treefile | 0.00132826 |
| cp__psbm.aln.fasta.treefile | 0.00885414 |
| cp__ndhb.aln.fasta.treefile | 0.00353529 |
| cp__clpp.aln.fasta.treefile | 0.0388675 |
| cp__ycf1.aln.fasta.treefile | 0.0234942 |
| cp__rpoc2.aln.fasta.treefile | 0.00547399 |
| cp__atph.aln.fasta.treefile | 0.00272465 |
| cp__rpoc1.aln.fasta.treefile | 0.00554604 |
| cp__ndhg.aln.fasta.treefile | 0.00918127 |
| cp__psbd.aln.fasta.treefile | 0.00121628 |
| cp__rpl16.aln.fasta.treefile | 0.00749722 |
| cp__matk.aln.fasta.treefile | 0.00906706 |
| cp__psbe.aln.fasta.treefile | 0.00076368 |
| cp__petd.aln.fasta.treefile | 0.00376119 |
| cp__rpl36.aln.fasta.treefile | 0.00665823 |
| cp__petl.aln.fasta.treefile | 0.0120217 |
| cp__rps4.aln.fasta.treefile | 0.00387198 |
| cp__ndhd.aln.fasta.treefile | 0.00329837 |
| cp__rps7.aln.fasta.treefile | 0.00261245 |
| cp__atpf.aln.fasta.treefile | 0.00286993 |
| cp__infa.aln.fasta.treefile | 0.0370038 |
| cp__rpl23.aln.fasta.treefile | 0.00777657 |
| cp__atpe.aln.fasta.treefile | 0.013261 |
| cp__rps8.aln.fasta.treefile | 0.00732274 |
| cp__ndhk.aln.fasta.treefile | 0.0145308 |
| cp__peta.aln.fasta.treefile | 0.00492338 |
| cp__petb.aln.fasta.treefile | 0.00612722 |
| cp__psbh.aln.fasta.treefile | 0.00496563 |
| cp__rpl33.aln.fasta.treefile | 0.00778801 |
| cp__rpl22.aln.fasta.treefile | 0.0268591 |
| cp__ndhi.aln.fasta.treefile | 0.00341644 |
| cp__psbn.aln.fasta.treefile | 0.00573106 |
| cp__atpb.aln.fasta.treefile | 0.00285453 |
| cp__psbc.aln.fasta.treefile | 0.00102038 |
| cp__cema.aln.fasta.treefile | 0.00719333 |
| cp__rps18.aln.fasta.treefile | 0.201974 |
| cp__rpl20.aln.fasta.treefile | 0.0125783 |
| cp__ndhj.aln.fasta.treefile | 0.00806609 |
| cp__rps15.aln.fasta.treefile | 0.0146804 |
| cp__psbi.aln.fasta.treefile | 0.0060973 |
| cp__psaj.aln.fasta.treefile | 0.0048523 |
| cp__rpoa.aln.fasta.treefile | 0.0227452 |
| cp__psbz.aln.fasta.treefile | 0.00424741 |
| cp__rbcl.aln.fasta.treefile | 0.00333535 |
| cp__psai.aln.fasta.treefile | 0.034108 |
| cp__rpl2.aln.fasta.treefile | 0.00183305 |
| cp__ndha.aln.fasta.treefile | 0.00744501 |
| cp__psbb.aln.fasta.treefile | 0.00402421 |
| cp__psac.aln.fasta.treefile | 0.00293536 |
| cp__rps19.aln.fasta.treefile | 0.0290225 |
| cp__rpl14.aln.fasta.treefile | 0.0045361 |
| cp__psab.aln.fasta.treefile | 0.00278157 |
| cp__psaa.aln.fasta.treefile | 0.00210408 |
| cp__rps16.aln.fasta.treefile | 0.0184891 |
| cp__psbl.aln.fasta.treefile | 0.002317 |
| cp__atpi.aln.fasta.treefile | 0.0118943 |
| cp__ndhh.aln.fasta.treefile | 0.00552578 |
| cp__ndhe.aln.fasta.treefile | 0.00526379 |
| cp__rps3.aln.fasta.treefile | 0.00755428 |
| mt__ccmfn.aln.fasta.treefile | 0.0200726 |
| mt__rps7.aln.fasta.treefile | 0.0125119 |
| mt__nad3.aln.fasta.treefile | 0.0213109 |
| mt__rps14.aln.fasta.treefile | 0.000833708 |
| mt__rps3.aln.fasta.treefile | 0.0132228 |
| mt__atp8.aln.fasta.treefile | 0.00583222 |
| mt__rpl2.aln.fasta.treefile | 0.00125532 |
| mt__rps10.aln.fasta.treefile | 0.00504704 |
| mt__rps1.aln.fasta.treefile | 0.0289891 |
| mt__nad1.aln.fasta.treefile | 0.00397987 |
| mt__matr.aln.fasta.treefile | 0.000643982 |
| mt__rpl16.aln.fasta.treefile | 0.000747042 |
| mt__rps13.aln.fasta.treefile | 0.00252502 |
| mt__rps4.aln.fasta.treefile | 0.0134491 |
| mt__nad2.aln.fasta.treefile | 0.00111093 |
| mt__cox3.aln.fasta.treefile | 0.0112636 |
| mt__ccmb.aln.fasta.treefile | 0.00976215 |
| mt__cox2.aln.fasta.treefile | 0.0352914 |
| mt__atp6.aln.fasta.treefile | 0.238034 |
| mt__nad6.aln.fasta.treefile | 0.037284 |
| mt__cob.aln.fasta.treefile | 0.0578753 |
| mt__ccmc.aln.fasta.treefile | 0.279719 |
| mt__nad4l.aln.fasta.treefile | 0.00305042 |
| mt__rpl10.aln.fasta.treefile | 0.0261708 |
| mt__rps12.aln.fasta.treefile | 0.0173284 |
| mt__ccmfc.aln.fasta.treefile | 0.00494301 |
| mt__atp4.aln.fasta.treefile | 0.010831 |
| mt__mttb.aln.fasta.treefile | 0.0167777 |
| mt__nad7.aln.fasta.treefile | 0.000578955 |
| mt__nad4.aln.fasta.treefile | 0.000666419 |
| mt__sdh4.aln.fasta.treefile | 0.0938926 |
| mt__rps19.aln.fasta.treefile | 0.0041565 |
| mt__sdh3.aln.fasta.treefile | 0.0237098 |
| mt__atp1.aln.fasta.treefile | 0.0438167 |
| mt__cox1.aln.fasta.treefile | 0.0292814 |
| mt__nad5.aln.fasta.treefile | 0.000434597 |
| mt__rpl5.aln.fasta.treefile | 0.00193294 |
| mt__atp9.aln.fasta.treefile | 0.283937 |
| mt__nad9.aln.fasta.treefile | 0.00527103 |

**Supplementary table 4.** The root-to-tip variance of all gene trees used in the analysis.

| MoleculeType | GeneName | AlignmentLength | Likelihood | CorrectedLikelihood |
| --- | --- | --- | --- | --- |
| cp | accd | 4890 | -66839.1 | -13.66852761 |
| cp | atpa | 1587 | -50230 | -31.65091367 |
| cp | atpb | 1521 | -45627.6 | -29.99842209 |
| cp | atpe | 480 | -15438.3 | -32.163125 |
| cp | atpf | 687 | -20544.4 | -29.90451237 |
| cp | atph | 327 | -6267.4 | -19.16636086 |
| cp | atpi | 921 | -22654 | -24.59717698 |
| cp | ccsa | 1296 | -42522.7 | -32.81072531 |
| cp | cema | 1959 | -40339.9 | -20.5920878 |
| cp | clpp | 831 | -26433.6 | -31.80938628 |
| cp | infa | 351 | -6558.71 | -18.68578348 |
| cp | matk | 2196 | -83255.7 | -37.91243169 |
| cp | ndha | 1200 | -39733.1 | -33.11091667 |
| cp | ndhb | 1698 | -31568.1 | -18.59134276 |
| cp | ndhc | 477 | -11146.4 | -23.36771488 |
| cp | ndhd | 1671 | -57201.1 | -34.23165769 |
| cp | ndhe | 339 | -9419.34 | -27.78566372 |
| cp | ndhf | 2667 | -113505 | -42.55905512 |
| cp | ndhg | 654 | -21570.4 | -32.982263 |
| cp | ndhh | 1257 | -36276.6 | -28.85966587 |
| cp | ndhi | 654 | -18173.3 | -27.78792049 |
| cp | ndhj | 603 | -14742.8 | -24.44908789 |
| cp | ndhk | 1140 | -26447 | -23.19912281 |
| cp | peta | 1056 | -31992.2 | -30.29564394 |
| cp | petb | 771 | -19151.9 | -24.84033722 |
| cp | petd | 810 | -15513.3 | -19.15222222 |
| cp | petg | 114 | -2660.85 | -23.34078947 |
| cp | petl | 183 | -3557.35 | -19.43907104 |
| cp | petn | 129 | -1947.88 | -15.09984496 |
| cp | psaa | 2334 | -55357.7 | -23.71795201 |
| cp | psab | 2208 | -55404.4 | -25.09257246 |
| cp | psac | 246 | -7013.42 | -28.5098374 |
| cp | psai | 153 | -3949.13 | -25.81130719 |
| cp | psaj | 180 | -4614.59 | -25.63661111 |
| cp | psba | 1080 | -21540.1 | -19.94453704 |
| cp | psbb | 1545 | -43270.6 | -28.00686084 |
| cp | psbc | 1467 | -36689.2 | -25.00967962 |
| cp | psbd | 1080 | -23237.2 | -21.51592593 |
| cp | psbe | 264 | -5099.01 | -19.31443182 |
| cp | psbf | 150 | -2432.87 | -16.21913333 |
| cp | psbh | 348 | -8053.01 | -23.14083333 |
| cp | psbi | 174 | -3327.65 | -19.12442529 |
| cp | psbj | 123 | -3245.95 | -26.3898374 |
| cp | psbk | 219 | -6337.82 | -28.93981735 |
| cp | psbl | 531 | -2649.06 | -4.988813559 |
| cp | psbm | 117 | -2979.21 | -25.46333333 |
| cp | psbn | 132 | -3037.8 | -23.01363636 |
| cp | psbt | 132 | -3548.44 | -26.88212121 |
| cp | psbz | 189 | -4653.34 | -24.62084656 |
| cp | rbcl | 1575 | -44957 | -28.54412698 |
| cp | rpl14 | 429 | -11962.8 | -27.88531469 |
| cp | rpl16 | 519 | -16389.4 | -31.57880539 |
| cp | rpl20 | 780 | -18031.9 | -23.11782051 |
| cp | rpl22 | 855 | -23897.3 | -27.95005848 |
| cp | rpl23 | 363 | -7623.83 | -21.0022865 |
| cp | rpl2 | 990 | -21958.9 | -22.18070707 |
| cp | rpl32 | 330 | -11401.9 | -34.55121212 |
| cp | rpl33 | 267 | -7628.16 | -28.56988764 |
| cp | rpl36 | 123 | -3592.68 | -29.20878049 |
| cp | rpoa | 1338 | -41184.8 | -30.78086697 |
| cp | rpob | 3786 | -109046 | -28.80243001 |
| cp | rpoc1 | 2487 | -73247.7 | -29.4522316 |
| cp | rpoc2 | 7239 | -190550 | -26.32269651 |
| cp | rps11 | 507 | -16154.2 | -31.86232742 |
| cp | rps12 | 495 | -8876.13 | -17.93157576 |
| cp | rps14 | 324 | -10250.3 | -31.6367284 |
| cp | rps15 | 435 | -12480.9 | -28.69172414 |
| cp | rps16 | 315 | -6651.72 | -21.11657143 |
| cp | rps18 | 1401 | -15274 | -10.90221271 |
| cp | rps19 | 459 | -10421.8 | -22.70544662 |
| cp | rps2 | 801 | -24488.6 | -30.57253433 |
| cp | rps3 | 1062 | -29157.6 | -27.45536723 |
| cp | rps4 | 756 | -21350.4 | -28.24126984 |
| cp | rps7 | 579 | -10699.3 | -18.47892919 |
| cp | rps8 | 474 | -16559.6 | -34.93586498 |
| cp | ycf1 | 15540 | -337962 | -21.74787645 |
| cp | ycf2 | 13077 | -243182 | -18.5961612 |
| cp | ycf3 | 552 | -12024.8 | -21.78405797 |
| cp | ycf4 | 807 | -22121 | -27.41140025 |
| mt | atp1 | 2250 | -36770 | -16.34222222 |
| mt | atp4 | 885 | -15532 | -17.55028249 |
| mt | atp6 | 2517 | -48839.2 | -19.4037346 |
| mt | atp8 | 855 | -16069 | -18.79415205 |
| mt | atp9 | 597 | -10389.6 | -17.40301508 |
| mt | ccmb | 879 | -9751.79 | -11.09418658 |
| mt | ccmc | 1323 | -18692.1 | -14.12857143 |
| mt | ccmfc | 2715 | -32303.9 | -11.89830571 |
| mt | ccmfn | 3219 | -38173.5 | -11.85880708 |
| mt | cob | 2442 | -23700.5 | -9.705364455 |
| mt | cox1 | 2682 | -28997.6 | -10.81193139 |
| mt | cox2 | 1875 | -26529.1 | -14.14885333 |
| mt | cox3 | 936 | -12784.9 | -13.6590812 |
| mt | matr | 2577 | -27156.9 | -10.53818393 |
| mt | mttb | 1125 | -19781.5 | -17.58355556 |
| mt | nad1 | 1152 | -19834.2 | -17.2171875 |
| mt | nad2 | 2525 | -21287.8 | -8.430811881 |
| mt | nad3 | 480 | -5889.18 | -12.269125 |
| mt | nad4 | 1803 | -22468.1 | -12.4615086 |
| mt | nad4l | 417 | -4891.5 | -11.73021583 |
| mt | nad5 | 2304 | -29785 | -12.92751736 |
| mt | nad6 | 1896 | -20280.4 | -10.6964135 |
| mt | nad7 | 1470 | -12992.7 | -8.838571429 |
| mt | nad9 | 1140 | -10095.5 | -8.855701754 |
| mt | rpl10 | 906 | -9263.54 | -10.22465784 |
| mt | rpl16 | 645 | -6028.32 | -9.346232558 |
| mt | rpl2 | 2571 | -23639 | -9.194476857 |
| mt | rpl5 | 855 | -11608.7 | -13.5774269 |
| mt | rps10 | 573 | -5493.77 | -9.587731239 |
| mt | rps12 | 672 | -7228.06 | -10.75604167 |
| mt | rps13 | 489 | -6159.98 | -12.59709611 |
| mt | rps14 | 477 | -3722.22 | -7.803396226 |
| mt | rps19 | 438 | -4515.22 | -10.30872146 |
| mt | rps1 | 1812 | -20440.6 | -11.28068433 |
| mt | rps3 | 4065 | -47691.3 | -11.73217712 |
| mt | rps4 | 2112 | -23235.7 | -11.00175189 |
| mt | rps7 | 840 | -8406.03 | -10.00717857 |
| mt | sdh3 | 945 | -9976.89 | -10.55755556 |
| mt | sdh4 | 831 | -11231 | -13.51504212 |

**Supplementary table 5.** The likelihood value that each gene contributed to the overall likelihood of the COMB-Proportional tree. The length of the gene alignment and the likelihood divided by the alignment length (corrected likelihood) are also presented.

| Topology1 | Topology2 | RF-Distance |
| --- | --- | --- |
| COMB-MERGED:COMB-Linked:COMB-Proportional:COMB-Unpartitioned | PLAST-Linked:PLAST-Propotional | 10 |
| COMB-MERGED:COMB-Linked:COMB-Proportional:COMB-Unpartitioned | COMB-Unlinked | 6 |
| COMB-MERGED:COMB-Linked:COMB-Proportional:COMB-Unpartitioned | PLAST-Unlinked | 24 |
| COMB-MERGED:COMB-Linked:COMB-Proportional:COMB-Unpartitioned | PLAST-Unpartitioned | 12 |
| COMB-MERGED:COMB-Linked:COMB-Proportional:COMB-Unpartitioned | MITO-Linked:MITO-Unpartitioned | 72 |
| COMB-MERGED:COMB-Linked:COMB-Proportional:COMB-Unpartitioned | MITO-Unlinked | 76 |
| COMB-MERGED:COMB-Linked:COMB-Proportional:COMB-Unpartitioned | MITO-Proportional | 70 |
| COMB-Unlinked | PLAST-Linked:PLAST-Propotional | 16 |
| COMB-Unlinked | PLAST-Unlinked | 18 |
| COMB-Unlinked | PLAST-Unpartitioned | 18 |
| COMB-Unlinked | MITO-Linked:MITO-Unpartitioned | 72 |
| COMB-Unlinked | MITO-Proportional | 70 |
| PLAST-Linked:PLAST-Propotional | PLAST-Unlinked | 14 |
| COMB-Unlinked | MITO-Unlinked | 76 |
| PLAST-Linked:PLAST-Propotional | MITO-Linked:MITO-Unpartitioned | 80 |
| PLAST-Linked:PLAST-Propotional | PLAST-Unpartitioned | 2 |
| PLAST-Linked:PLAST-Propotional | MITO-Proportional | 78 |
| PLAST-Linked:PLAST-Propotional | MITO-Unlinked | 84 |
| PLAST-Unlinked | PLAST-Unpartitioned | 14 |
| PLAST-Unlinked | MITO-Linked:MITO-Unpartitioned | 86 |
| PLAST-Unlinked | MITO-Proportional | 84 |
| PLAST-Unlinked | MITO-Unlinked | 92 |
| PLAST-Unpartitioned | MITO-Linked:MITO-Unpartitioned | 82 |
| PLAST-Unpartitioned | MITO-Proportional | 80 |
| PLAST-Unpartitioned | MITO-Unlinked | 86 |
| MITO-Linked:MITO-Unpartitioned | MITO-Proportional | 8 |
| MITO-Linked:MITO-Unpartitioned | MITO-Unlinked | 28 |
| MITO-Proportional | MITO-Unlinked | 24 |

**Supplementary table 6.** The Robinson-Foulds distances, as estimated using all edges, between the COMB, PLAST and MITO trees for all approaches.

| Topology1 | Topology2 | RF-Distance |
| --- | --- | --- |
| COMB-MERGED | COMB-Linked | 10 |
| COMB-MERGED | COMB-Proportional | 6 |
| COMB-MERGED | COMB-Unlinked | 4 |
| COMB-MERGED | PLAST-Linked | 12 |
| COMB-MERGED | COMB-Unpartitioned | 2 |
| COMB-MERGED | PLAST-Proportional | 14 |
| COMB-MERGED | PLAST-Unlinked | 22 |
| COMB-MERGED | PLAST-Linked | 12 |
| COMB-MERGED | MITO-Partitioned | 74 |
| COMB-MERGED | MITO-Unlinked | 88 |
| COMB-MERGED | MITO-Proportional | 78 |
| COMB-MERGED | MITO-Unpartitioned | 74 |
| COMB-Linked | COMB-Proportional | 8 |
| COMB-Linked | COMB-Unlinked | 4 |
| COMB-Linked | COMB-Unpartitioned | 2 |
| COMB-Linked | PLAST-Linked | 12 |
| COMB-Linked | PLAST-Proportional | 14 |
| COMB-Linked | PLAST-Unlinked | 18 |
| COMB-Linked | PLAST-Linked | 14 |
| COMB-Linked | MITO-Partitioned | 70 |
| COMB-Linked | MITO-Proportional | 74 |
| COMB-Linked | MITO-Unlinked | 82 |
| COMB-Linked | MITO-Unpartitioned | 68 |
| COMB-Proportional | COMB-Unlinked | 4 |
| COMB-Proportional | COMB-Unpartitioned | 2 |
| COMB-Proportional | PLAST-Linked | 12 |
| COMB-Proportional | PLAST-Proportional | 14 |
| COMB-Proportional | PLAST-Unlinked | 22 |
| COMB-Proportional | PLAST-Linked | 12 |
| COMB-Proportional | MITO-Partitioned | 70 |
| COMB-Proportional | MITO-Proportional | 74 |
| COMB-Proportional | MITO-Unlinked | 84 |
| COMB-Proportional | MITO-Unpartitioned | 70 |
| COMB-Unlinked | COMB-Unpartitioned | 12 |
| COMB-Unlinked | PLAST-Linked | 22 |
| COMB-Unlinked | PLAST-Proportional | 26 |
| COMB-Unlinked | PLAST-Unlinked | 26 |
| COMB-Unlinked | PLAST-Linked | 24 |
| COMB-Unlinked | MITO-Partitioned | 82 |
| COMB-Unlinked | MITO-Proportional | 86 |
| COMB-Unlinked | MITO-Unlinked | 96 |
| COMB-Unlinked | MITO-Unpartitioned | 80 |
| COMB-Unpartitioned | PLAST-Linked | 14 |
| COMB-Unpartitioned | PLAST-Proportional | 16 |
| COMB-Unpartitioned | PLAST-Unlinked | 24 |
| COMB-Unpartitioned | PLAST-Linked | 16 |
| COMB-Unpartitioned | MITO-Partitioned | 78 |
| COMB-Unpartitioned | MITO-Proportional | 82 |
| COMB-Unpartitioned | MITO-Unlinked | 90 |
| COMB-Unpartitioned | MITO-Unpartitioned | 76 |
| PLAST-Linked | PLAST-Unlinked | 12 |
| PLAST-Linked | PLAST-Proportional | 4 |
| PLAST-Linked | MITO-Partitioned | 78 |
| PLAST-Linked | PLAST-Linked | 8 |
| PLAST-Linked | MITO-Proportional | 82 |
| PLAST-Linked | MITO-Unlinked | 90 |
| PLAST-Linked | MITO-Unpartitioned | 76 |
| PLAST-Proportional | PLAST-Unlinked | 10 |
| PLAST-Proportional | PLAST-Linked | 4 |
| PLAST-Proportional | MITO-Partitioned | 80 |
| PLAST-Proportional | MITO-Proportional | 84 |
| PLAST-Proportional | MITO-Unlinked | 92 |
| PLAST-Proportional | MITO-Unpartitioned | 78 |
| PLAST-Unlinked | MITO-Partitioned | 80 |
| PLAST-Unlinked | PLAST-Linked | 10 |
| PLAST-Unlinked | MITO-Proportional | 84 |
| PLAST-Unlinked | MITO-Unlinked | 92 |
| PLAST-Unlinked | MITO-Unpartitioned | 78 |
| PLAST-Linked | MITO-Partitioned | 78 |
| PLAST-Linked | MITO-Unlinked | 90 |
| PLAST-Linked | MITO-Proportional | 80 |
| PLAST-Linked | MITO-Unpartitioned | 76 |
| MITO-Partitioned | MITO-Proportional | 16 |
| MITO-Partitioned | MITO-Unlinked | 32 |
| MITO-Partitioned | MITO-Unpartitioned | 8 |
| MITO-Proportional | MITO-Unlinked | 24 |
| MITO-Proportional | MITO-Unpartitioned | 14 |
| MITO-Unlinked | MITO-Unpartitioned | 10 |

**Supplementary table 7.** The Robinson-Foulds distances, as estimated using only well supported edges (≥95% UFBoot), between the COMB, PLAST and MITO trees for all approaches.

| org | V1 | n | genes |
| --- | --- | --- | --- |
| cp | cluster0 | 1 | accd |
| cp | cluster1 | 10 | atpa atpb atpi ndhc ndhj peta psac psbh psbk ycf3 |
| cp | cluster10 | 1 | ndhf |
| cp | cluster11 | 2 | ndhg ycf4 |
| cp | cluster12 | 3 | ndhh rpl14 rpl36 |
| cp | cluster13 | 4 | ndhk psbt rpl16 rps16 |
| cp | cluster14 | 3 | petb petd rbcl |
| cp | cluster15 | 8 | petg psbe psbf psbi psbj psbl psbn psbz |
| mt | cluster16 | -5 | ccmb nad2 nad3 nad4 nad4l |
| cp | cluster16 | 1 | petn |
| cp | cluster17 | 1 | psba |
| cp | cluster18 | 3 | rpl22 rpl32 rps18 |
| mt | cluster19 | -1 | rps19 |
| cp | cluster19 | 3 | rpl23 rpl2 rps7 |
| cp | cluster2 | 9 | atpe atpf rpl33 rpob rpoc1 rps11 rps14 rps2 rps4 |
| mt | cluster20 | -2 | atp1 rps10 |
| cp | cluster20 | 1 | rps12 |
| cp | cluster21 | 4 | rps15 rps19 rps3 rps8 |
| cp | cluster22 | 1 | ycf1 |
| cp | cluster23 | 1 | ycf2 |
| mt | cluster24 | -5 | atp4 atp8 rpl10 rps3 rps4 |
| mt | cluster25 | -1 | atp6 |
| mt | cluster26 | -4 | atp9 cox2 nad6 sdh4 |
| mt | cluster27 | -3 | ccmc mttb sdh3 |
| mt | cluster28 | -2 | ccmfc ccmfn |
| mt | cluster29 | -3 | cob cox1 nad1 |
| cp | cluster3 | 6 | atph psaa psab psbb psbc psbd |
| mt | cluster30 | -2 | cox3 nad5 |
| mt | cluster31 | -1 | matr |
| mt | cluster32 | -1 | nad7 |
| mt | cluster33 | -3 | nad9 rpl16 rps12 |
| mt | cluster34 | -1 | rpl2 |
| mt | cluster35 | -4 | rpl5 rps13 rps14 rps7 |
| mt | cluster36 | -1 | rps1 |
| cp | cluster4 | 3 | ccsa cema rpl20 |
| cp | cluster5 | 1 | clpp |
| cp | cluster6 | 3 | infa rpoa rpoc2 |
| cp | cluster7 | 1 | matk |
| cp | cluster8 | 8 | ndha ndhd ndhe ndhi petl psai psaj psbm |
| cp | cluster9 | 1 | ndhb |

## Supplementary Table 8. Individual genes from the organelle genomes and the cluster with which they are found based on PartitionFinder. Negative numbers correspond to Mitochondrial genes.

| organelle | gene_name | checked | file | modelshifts | rootmodel | clade2 | clade3 | clade4 | clade5 | clade6 | clade7 | clade8 |
| --- | --- | --- | --- | --- | --- | --- | --- | --- | --- | --- | --- | --- |
| cp | accd | T | cp__accd.aln.fastaNewick.tre.gophy.results.tre | 3 | Tracheophyta | rosids | Mesangiospermae | Anthocerotophyta;Bryophyta;Marchantiophyta |  |  |  |  |
| cp | atpa | T | cp__atpa.aln.fastaNewick.tre.gophy.results.tre | 2 | all | Tracheophyta | Anthocerotophyta;Bryophyta;Marchantiophyta |  |  |  |  |  |
| cp | atpb | T | cp__atpb.aln.fastaNewick.tre.gophy.results.tre | 1 | Anthocerotophyta;Bryophyta;Marchantiophyta |  |  |  |  |  |  |  |
| cp | atpe | T | cp__atpe.aln.fastaNewick.tre.gophy.results.tre | 1 | Anthocerotophyta;Bryophyta;Marchantiophyta |  |  |  |  |  |  |  |
| cp | atpf | T | cp__atpf.aln.fastaNewick.tre.gophy.results.tre | 1 | Mesangiospermae |  |  |  |  |  |  |  |
| cp | atph | T | cp__atph.aln.fastaNewick.tre.gophy.results.tre | 2 | Austrobaileyales;Mesangiospermae | Anthocerotophyta;Bryophyta;Marchantiophyta |  |  |  |  |  |  |
| cp | atpi | T | cp__atpi.aln.fastaNewick.tre.gophy.results.tre | 1 | Anthocerotophyta;Bryophyta;Marchantiophyta |  |  |  |  |  |  |  |
| cp | ccsa | T | cp__ccsa.aln.fastaNewick.tre.gophy.results.tre | 2 | Hepatica_maxima;Apium_graveolens | Angiosperms |  |  |  |  |  |  |
| cp | cema | T | cp__cema.aln.fastaNewick.tre.gophy.results.tre | 3 | Solanoideae | Angiosperms | Tracheophyta |  |  |  |  |  |
| cp | clpp | T | cp__clpp.aln.fastaNewick.tre.gophy.results.tre | 7 | Tracheophyta | Anthocerotophyta;Bryophyta;Marchantiophyta | Spermatophyta | core eudicots | Solanum;Capsicum | Senna_occidentalis;Leucaena_trichandra | Malvales |  |
| cp | infa | T | cp__infa.aln.fastaNewick.tre.gophy.results.tre | 2 | Tracheophyta | core eudicots | Anthocerotophyta;Bryophyta;Marchantiophyta |  |  |  |  |  |
| cp | matk | T | cp__matk.aln.fastaNewick.tre.gophy.results.tre | 2 | Tracheophyta | monocots;eudicots | Anthocerotophyta;Bryophyta;Marchantiophyta |  |  |  |  |  |
| cp | ndha | T | cp__ndha.aln.fastaNewick.tre.gophy.results.tre | 2 | Tracheophyta | Mesangiospermae | Anthocerotophyta;Bryophyta;Marchantiophyta |  |  |  |  |  |
| cp | ndhb | T | cp__ndhb.aln.fastaNewick.tre.gophy.results.tre | 1 | all | Solanum |  |  |  |  |  |  |
| cp | ndhc | T | cp__ndhc.aln.fastaNewick.tre.gophy.results.tre | 2 | Tracheophyta | Angiosperms | Anthocerotophyta;Bryophyta;Marchantiophyta |  |  |  |  |  |
| cp | ndhd | T | cp__ndhd.aln.fastaNewick.tre.gophy.results.tre | 2 | Tracheophyta | Mesangiospermae | Anthocerotophyta;Bryophyta;Marchantiophyta |  |  |  |  |  |
| cp | ndhe | T | cp__ndhe.aln.fastaNewick.tre.gophy.results.tre | 1 | all | Angiosperms |  |  |  |  |  |  |
| cp | ndhf | T | cp__ndhf.aln.fastaNewick.tre.gophy.results.tre | 3 | Tracheophyta | Spermatophyta | Anthocerotophyta;Bryophyta;Marchantiophyta | Mesangiospermae |  |  |  |  |
| cp | ndhg | T | cp__ndhg.aln.fastaNewick.tre.gophy.results.tre | 3 | Tracheophyta | Gossypium_arboreum;Gossypium_barbadense | core eudicots | Anthocerotophyta;Bryophyta;Marchantiophyta |  |  |  |  |
| cp | ndhh | T | cp__ndhh.aln.fastaNewick.tre.gophy.results.tre | 2 | Tracheophyta | Angiosperms | Anthocerotophyta;Bryophyta;Marchantiophyta |  |  |  |  |  |
| cp | ndhi | T | cp__ndhi.aln.fastaNewick.tre.gophy.results.tre | 1 | all | eudicots |  |  |  |  |  |  |
| cp | ndhj | T | cp__ndhj.aln.fastaNewick.tre.gophy.results.tre | 1 | all | Solanoideae |  |  |  |  |  |  |
| cp | ndhk | T | cp__ndhk.aln.fastaNewick.tre.gophy.results.tre | 1 | all | Bryophytina |  |  |  |  |  |  |
| cp | peta | T | cp__peta.aln.fastaNewick.tre.gophy.results.tre | 2 | Tracheophyta | Anthocerotophyta;Bryophyta;Marchantiophyta | Apium_graveolens;Osmanthus_fragrans |  |  |  |  |  |
| cp | petb | T | cp__petb.aln.fastaNewick.tre.gophy.results.tre | 1 | Tracheophyta | Anthocerotophyta;Bryophyta;Marchantiophyta |  |  |  |  |  |  |
| cp | petd | T | cp__petd.aln.fastaNewick.tre.gophy.results.tre | 1 | all | Brassica_juncea;Raphanus_sativus |  |  |  |  |  |  |
| cp | petl | T | cp__petl.aln.fastaNewick.tre.gophy.results.tre | 0 | all |  |  |  |  |  |  |  |
| cp | petn | T | cp__petn.aln.fastaNewick.tre.gophy.results.tre | 0 | all |  |  |  |  |  |  |  |
| cp | psaa | T | cp__psaa.aln.fastaNewick.tre.gophy.results.tre | 2 | Tracheophyta | Anthocerotophyta;Bryophyta;Marchantiophyta | Spermatophyta |  |  |  |  |  |
| cp | psab | T | cp__psab.aln.fastaNewick.tre.gophy.results.tre | 2 | Tracheophyta | Anthocerotophyta;Bryophyta;Marchantiophyta | Marchantiales |  |  |  |  |  |
| cp | psac | T | cp__psac.aln.fastaNewick.tre.gophy.results.tre | 2 | Tracheophyta | Anthocerotophyta;Bryophyta;Marchantiophyta | Marchantiales |  |  |  |  |  |
| cp | psai | T | cp__psai.aln.fastaNewick.tre.gophy.results.tre | 1 | all | Spermatophyta |  |  |  |  |  |  |
| cp | psaj | T | cp__psaj.aln.fastaNewick.tre.gophy.results.tre | 0 | all |  |  |  |  |  |  |  |
| cp | psba | T | cp__psba.aln.fastaNewick.tre.gophy.results.tre | 1 | all | Mesangiospermae |  |  |  |  |  |  |
| cp | psbb | T | cp__psbb.aln.fastaNewick.tre.gophy.results.tre | 2 | Tracheophyta | Spermatophyta | Anthocerotophyta;Bryophyta;Marchantiophyta |  |  |  |  |  |
| cp | psbc | T | cp__psbc.aln.fastaNewick.tre.gophy.results.tre | 2 | Tracheophyta | Spermatophyta | Anthocerotophyta;Bryophyta;Marchantiophyta |  |  |  |  |  |
| cp | psbd | T | cp__psbd.aln.fastaNewick.tre.gophy.results.tre | 2 | Tracheophyta | Anthocerotophyta;Bryophyta;Marchantiophyta | Angiosperms |  |  |  |  |  |
| cp | psbe | T | cp__psbe.aln.fastaNewick.tre.gophy.results.tre | 1 | all | Bryophytina |  |  |  |  |  |  |
| cp | psbf | T | cp__psbf.aln.fastaNewick.tre.gophy.results.tre | 4 | all | Bidens_bipinnata;Diplostephium_hartwegii | Solanales | Ammopiptanthus_nanus;Prunus_salicina_x_Prunus_armeniaca | Brassica_carinata;Arabis_alpina |  |  |  |
| cp | psbh | T | cp__psbh.aln.fastaNewick.tre.gophy.results.tre | 1 | Tracheophyta | Anthocerotophyta;Bryophyta;Marchantiophyta |  |  |  |  |  |  |
| cp | psbi | T | cp__psbi.aln.fastaNewick.tre.gophy.results.tre | 1 | all | Angiosperms |  |  |  |  |  |  |
| cp | psbj | T | cp__psbj.aln.fastaNewick.tre.gophy.results.tre | 1 | all | Angiosperms |  |  |  |  |  |  |
| cp | psbk | T | cp__psbk.aln.fastaNewick.tre.gophy.results.tre | 1 | all | Mesangiospermae |  |  |  |  |  |  |
| cp | psbl | T | cp__psbl.aln.fastaNewick.tre.gophy.results.tre | 0 | all |  |  |  |  |  |  |  |
| cp | psbm | T | cp__psbm.aln.fastaNewick.tre.gophy.results.tre | 1 | all | Haematoxylum_brasiletto;Acacia_ligulata |  |  |  |  |  |  |
| cp | psbn | T | cp__psbn.aln.fastaNewick.tre.gophy.results.tre | 1 | all | Bidens_bipinnata;Diplostephium_hartwegii |  |  |  |  |  |  |
| cp | psbt | T | cp__psbt.aln.fastaNewick.tre.gophy.results.tre | 1 | all | Callicladium_imponens;Ptychomnion_cygnisetum |  |  |  |  |  |  |
| cp | psbz | T | cp__psbz.aln.fastaNewick.tre.gophy.results.tre | 0 | all |  |  |  |  |  |  |  |
| cp | rbcl | T | cp__rbcl.aln.fastaNewick.tre.gophy.results.tre | 2 | Tracheophyta | Anthocerotophyta;Bryophyta;Marchantiophyta | Angiosperms |  |  |  |  |  |
| cp | rpl2 | T | cp__rpl2.aln.fastaNewick.tre.gophy.results.tre | 3 | all | Bidens_bipinnata;Helianthus_tuberosus | Bryanae | Haematoxylum_brasilett:Acacia_ligulata |  |  |  |  |
| cp | rpl14 | T | cp__rpl14.aln.fastaNewick.tre.gophy.results.tre | 1 | all | core eudicots |  |  |  |  |  |  |
| cp | rpl16 | T | cp__rpl16.aln.fastaNewick.tre.gophy.results.tre | 1 | all | core eudicots |  |  |  |  |  |  |
| cp | rpl20 | T | cp__rpl20.aln.fastaNewick.tre.gophy.results.tre | 2 | all | eudicots | Ammopiptanthus_nanus;Acacia_ligulata |  |  |  |  |  |
| cp | rpl22 | T | cp__rpl22.aln.fastaNewick.tre.gophy.results.tre | 1 | all | Austrobaileyales;Mesangiospermae |  |  |  |  |  |  |
| cp | rpl23 | T | cp__rpl23.aln.fastaNewick.tre.gophy.results.tre | 1 | all | Bidens |  |  |  |  |  |  |
| cp | rpl32 | T | cp__rpl32.aln.fastaNewick.tre.gophy.results.tre | 2 | all | Austrobaileyales;Mesangiospermae | core eudicots |  |  |  |  |  |
| cp | rpl33 | T | cp__rpl33.aln.fastaNewick.tre.gophy.results.tre | 0 | all |  |  |  |  |  |  |  |
| cp | rpl36 | T | cp__rpl36.aln.fastaNewick.tre.gophy.results.tre | 2 | all | Spermatophyta | Bryophyta |  |  |  |  |  |
| cp | rpoa | T | cp__rpoa.aln.fastaNewick.tre.gophy.results.tre | 2 | all | Mesangiospermae | eudicots |  |  |  |  |  |
| cp | rpob | T | cp__rpob.aln.fastaNewick.tre.gophy.results.tre | 2 | Tracheophyta | Anthocerotophyta;Bryophyta;Marchantiophyta | core eudicots |  |  |  |  |  |
| cp | rpoc1 | T | cp__rpoc1.aln.fastaNewick.tre.gophy.results.tre | 3 | Tracheophyta | Anthocerotophyta;Bryophyta;Marchantiophyta | Angiosperms | Gossypium_arboreum;Gossypium_barbadense |  |  |  |  |
| cp | rpoc2 | T | cp__rpoc2.aln.fastaNewick.tre.gophy.results.tre | 3 | Tracheophyta | Austrobaileyales;Mesangiospermae | core eudicots | Anthocerotophyta;Bryophyta;Marchantiophyta |  |  |  |  |
| cp | rps11 | T | cp__rps11.aln.fastaNewick.tre.gophy.results.tre | 0 | all |  |  |  |  |  |  |  |
| cp | rps12 | T | cp__rps12.aln.fastaNewick.tre.gophy.results.tre | 1 | Tracheophyta | Anthocerotophyta;Bryophyta;Marchantiophyta |  |  |  |  |  |  |
| cp | rps14 | T | cp__rps14.aln.fastaNewick.tre.gophy.results.tre | 1 | Tracheophyta | Anthocerotophyta;Bryophyta;Marchantiophyta |  |  |  |  |  |  |
| cp | rps15 | T | cp__rps15.aln.fastaNewick.tre.gophy.results.tre | 1 | all | proteales;core eudicots |  |  |  |  |  |  |
| cp | rps16 | T | cp__rps16.aln.fastaNewick.tre.gophy.results.tre | 1 | all | Mesangiospermae |  |  |  |  |  |  |
| cp | rps18 | T | cp__rps18.aln.fastaNewick.tre.gophy.results.tre | 1 | all | Brassica_carinata;Arabis_alpina |  |  |  |  |  |  |
| cp | rps19 | T | cp__rps19.aln.fastaNewick.tre.gophy.results.tre | 1 | all | eudicots |  |  |  |  |  |  |
| cp | rps2 | T | cp__rps2.aln.fastaNewick.tre.gophy.results.tre | 0 | all |  |  |  |  |  |  |  |
| cp | rps3 | T | cp__rps3.aln.fastaNewick.tre.gophy.results.tre | 1 | all | Austrobaileyales;Mesangiospermae |  |  |  |  |  |  |
| cp | rps4 | T | cp__rps4.aln.fastaNewick.tre.gophy.results.tre | 2 | Tracheophyta | Anthocerotophyta;Bryophyta;Marchantiophyta | Bryophytina |  |  |  |  |  |
| cp | rps7 | T | cp__rps7.aln.fastaNewick.tre.gophy.results.tre | 1 | all | Austrobaileyales;Mesangiospermae |  |  |  |  |  |  |
| cp | rps8 | T | cp__rps8.aln.fastaNewick.tre.gophy.results.tre | 2 | all | Apium_graveolens;Osmanthus_fragrans | core eudicots |  |  |  |  |  |
| cp | ycf1 | T | cp__ycf1.aln.fastaNewick.tre.gophy.results.tre | 3 | all | Spermatophyta | Jungermanniopsida | Mesangiospermae |  |  |  |  |
| cp | ycf2 | T | cp__ycf2.aln.fastaNewick.tre.gophy.results.tre | 3 | Tracheophyta | Anthocerotophyta;Bryophyta;Marchantiophyta | Spermatophyta | Austrobaileyales;Mesangiospermae |  |  |  |  |
| cp | ycf3 | T | cp__ycf3.aln.fastaNewick.tre.gophy.results.tre | 0 | all |  |  |  |  |  |  |  |
| cp | ycf4 | T | cp__ycf4.aln.fastaNewick.tre.gophy.results.tre | 3 | Tracheophyta | Anthocerotophyta;Bryophyta;Marchantiophyta | Angiosperms | Papilionoideae |  |  |  |  |

**Supplementary table 9.** All the chloroplast genes, and the location on the PLAST-Proportional topology of their inferred model shift(s). The root model is where the first, if any model shift is inferred to have occurred.

| organelle | gene_name | checked | modelshifts | rootmodel | clade2 | clade3 | clade4 | clade5 | clade6 | clade7 | clade8 |
| --- | --- | --- | --- | --- | --- | --- | --- | --- | --- | --- | --- |
| mt | atp1 | T | 4 |  | Bryophyta;Marchantiophyta | Anthocerotophyta;Bryophyta;Marchantiophyta | Angiosperms | Brassica_juncea;Brassica_rapa |  |  |  |
| mt | atp4 | T | 4 |  | Anthocerotophyta;Bryophyta;Marchantiophyta | Gymnosperms | Malvaceae | Ajuga_reptans;Hesperelaea_palmeri |  |  |  |
| mt | atp6 | T | 2 |  | Spermatophyta | Mesangiospermae |  |  |  |  |  |
| mt | atp8 | T | 3 |  | Anthocerotophyta;Bryophyta;Marchantiophyta | Angiosperms | fabids |  |  |  |  |
| mt | atp9 | T | 1 |  | Austrobaileyales;Mesangiospermae |  |  |  |  |  |  |
| mt | ccmb | T | 2 |  | Bryophyta | Angiosperms |  |  |  |  |  |
| mt | ccmc | T | 2 |  | Anthocerotophyta;Bryophyta;Marchantiophyta | Angiosperms |  |  |  |  |  |
| mt | ccmfc | T | 4 |  | Bryophyta | Anthocerotophyta;Bryophyta;Marchantiophyta | Angiosperms | Bombax_ceiba;Hibiscus_cannabinus |  |  |  |
| mt | ccmfn | T | 4 |  | Spermatophyta | Angiosperms | Anthocerotophyta;Bryophyta;Marchantiophyta | Bryophyta |  |  |  |
| mt | cob | T | 2 |  | Angiosperms | Anthocerotophyta;Bryophyta;Marchantiophyta |  |  |  |  |  |
| mt | cox1 | T | 2 |  | Anthocerotophyta;Bryophyta;Marchantiophyta | Mesangiospermae |  |  |  |  |  |
| mt | cox2 | T | 4 |  | Spermatophyta | Glycyrrhiza_uralensis;Mirabilis_jalapa | Macadamia_integrifolia;Vitis_vinifera | Mesangiospermae |  |  |  |
| mt | cox3 | T | 2 |  | Angiosperms | Bryophyta |  |  |  |  |  |
| mt | matr | T | 2 |  | Glycyrrhiza_uralensis;Mirabilis_jalapa | Papilionoideae |  |  |  |  |  |
| mt | mttb | T | 3 |  | Anthocerotophyta;Bryophyta;Marchantiophyta | Spermatophyta | Angiosperms |  |  |  |  |
| mt | nad1 | T | 2 |  | Anthocerotophyta;Bryophyta;Marchantiophyta | Angiosperms |  |  |  |  |  |
| mt | nad2 | t | 4 |  | Spermatophyta | Bryophyta | Eudicots | Angiosperms |  |  |  |
| mt | nad3 | T | 1 |  | Angiosperms |  |  |  |  |  |  |
| mt | nad4 | T | 1 |  | Angiosperms |  |  |  |  |  |  |
| mt | nad4l | T | 1 |  | Angiosperms |  |  |  |  |  |  |
| mt | nad5 | T | 3 |  | Bryophyta | Spermatophyta | Angiosperms |  |  |  |  |
| mt | nad6 | T | 4 |  | Spermatophyta | Austrobaileyales;Mesangiospermae | commelinids | Poaceae |  |  |  |
| mt | nad7 | T | 1 |  | Angiosperms |  |  |  |  |  |  |
| mt | nad9 | T | 3 |  | Anthocerotophyta;Bryophyta;Marchantiophyta | Angiosperms | Glycyrrhiza_uralensis;Ammopiptanthus_nanus |  |  |  |  |
| mt | rpl10 | T | 1 |  | Bryophyta |  |  |  |  |  |  |
| mt | rpl16 | T | 1 |  | Angiosperms |  |  |  |  |  |  |
| mt | rpl2 | T | 2 |  | Bryophyta | Angiosperms |  |  |  |  |  |
| mt | rpl5 | T | 3 |  | Bryophyta | Angiosperms | Gossypium_harknessii;Gossypium_raimondii |  |  |  |  |
| mt | rps1 | T | 1 |  | Callicladium_imponens;Sanionia_uncinata |  |  |  |  |  |  |
| mt | rps10 | T | 1 |  | Mesangiospermae |  |  |  |  |  |  |
| mt | rps12 | T | 3 |  | Bryophyta | Mesangiospermae | Core Caryophyllales |  |  |  |  |
| mt | rps13 | T | 1 |  | Angiosperms |  |  |  |  |  |  |
| mt | rps14 | T | 0 |  |  |  |  |  |  |  |  |
| mt | rps19 | T | 2 |  | Bryophyta;Marchantiophyta | Mesangiospermae |  |  |  |  |  |
| mt | rps3 | T | 3 |  | Gymnosperms | Austrobaileyales;Mesangiospermae | Anthocerotophyta;Bryophyta;Marchantiophyta |  |  |  |  |
| mt | rps4 | T | 4 |  | Gymnosperms | Mesangiospermae | Glycyrrhiza_uralensis;Lagerstroemia_indica | Anthocerotophyta;Bryophyta;Marchantiophyta |  |  |  |
| mt | rps7 | T | 1 |  | Angiosperms |  |  |  |  |  |  |
| mt | sdh3 | T | 2 |  | Anthocerotophyta;Bryophyta;Marchantiophyta | Angiosperms |  |  |  |  |  |
| mt | sdh4 | T | 1 |  | Angiosperms |  |  |  |  |  |  |

**Supplementary table 10.** All the mitochondrial genes, and the location on the MITO-Proportional topology of their inferred model shift(s). The root model is where the first, if any, model shift is inferred to have occurred.

## Supplementary Figures

**Supplementary Figure 1. Chloroplast gene occupancy data for the land plant taxa used in the study.** The y-axis lists the 226 land plant taxa in the study, and the x-axis has the total number of genes out of a possible 79 for each taxon. The bars are colored based on major body plans.

**Supplementary Figure 2. Mitochondrial gene occupancy data for the land plant taxa used in the study.** The y-axis lists the 226 land plant taxa in the study, and the x-axis has the total number of genes out of a possible 39 for each taxon. The bars are colored based on major body plans.

**Supplementary Figure 3. The COMB-Merged topology with chloroplast gene relationships mapped onto the nodes regardless of support.** The COMB-Merged topology, rooted on the Bryophytes, with the total number of concordant chloroplast genes mapped onto the nodes.

**Supplementary Figure 4. The COMB-Merged topology with well supported (≥95% UFBoot) chloroplast gene relationships mapped onto the nodes.** The COMB-Merged topology, rooted on the Bryophytes, with the total number of concordant and well supported (≥95% UFBoot) chloroplast genes mapped onto the nodes.

**Supplementary Figure 5. The PLAST-Proportional topology with chloroplast gene relationships mapped onto the nodes regardless of support.** The PLAST-Proportional topology, rooted on the Bryophytes, with the total number of concordant chloroplast genes mapped onto the nodes.

**Supplementary Figure 6. The PLAST-Proportional topology with well supported (≥95% UFBoot) chloroplast gene relationships mapped onto the nodes.** The PLAST-Proportional topology, rooted on the Bryophytes, with the total number of concordant and well supported (≥95% UFBoot) chloroplast genes mapped onto the nodes.

**Supplementary Figure 7. The COMB-Merged topology with mitochondrion gene relationships mapped onto the nodes regardless of support.** The COMB-Merged topology, rooted on the Bryophytes, with the total number of concordant mitochondrion genes mapped onto the nodes.

**Supplementary Figure 8. The COMB-Merged topology with well supported (≥95% UFBoot) mitochondrion gene relationships mapped onto the nodes.** The COMB-Merged topology, rooted on the Bryophytes, with the total number of concordant and well supported (≥95% UFBoot) mitochondrion genes mapped onto the nodes.

**Supplementary Figure 9. The MITO-Proportional topology with chloroplast gene relationships mapped onto the nodes regardless of support.** The MITO-Proportional topology, rooted on the Bryophytes, with the total number of concordant mitochondrion genes mapped onto the nodes.

**Supplementary Figure 10. The MITO-Proportional topology with well supported (≥95% UFBoot) mitochondrion gene relationships mapped onto the nodes.** The PLAST-Proportional topology, rooted on the Bryophytes, with the total number of concordant and well supported (≥95% UFBoot) mitochondrion genes mapped onto the nodes.

**Supplementary Figure 11. Predicted shifts in compositional heterogeneity across the PLAST-Proportional tree.** Labels on each node and different colors show the divergences at which a shift in compositional heterogeneity is predicted to occur based on the concatenated plastome supermatrix.

**Supplementary Figure 12. Predicted shifts in compositional heterogeneity across the MITO-Proportional tree.** Labels on each node and different colors show the divergences at which a shift in compositional heterogeneity is predicted to occur based on the concatenated mitochondrion supermatrix.

**Supplementary Figure 13. All relationships in the PLAST-Proportional tree with at least one gene predicted to have a shift in compositional heterogeneity at the relationship.** The x-axis contains the relationships in the PLAST-Proportional tree with an inferred shift in compositional heterogeneity; if the clade does not include a common or scientific name, the most distantly related taxa in the clade are used to denote it. The y-axis shows the percent of genes out of 78 that have an inferred shift at the relationship.

**Supplementary Figure 14. All relationships in the MITO-Proportional tree with at least one gene predicted to have a shift in compositional heterogeneity at the relationship.** The x-axis contains the relationships in the MITO-Proportional tree with an inferred shift in compositional heterogeneity; if the clade does not include a common or scientific name, the most distantly related taxa in the clade are used to denote it. The y-axis shows the percent of genes out of 38 that have an inferred shift at the relationship.
